# Detrimental *NFKB1* missense variants affecting the Rel-homology domain of p105/p50

**DOI:** 10.3389/fimmu.2022.965326

**Published:** 2022-08-29

**Authors:** Manfred Fliegauf, Matias Kinnunen, Sara Posadas-Cantera, Nadezhda Camacho-Ordonez, Hassan Abolhassani, Laia Alsina, Faranaz Atschekzei, Delfien J. Bogaert, Siobhan O. Burns, Joseph A. Church, Gregor Dückers, Alexandra F. Freeman, Lennart Hammarström, Leif Gunnar Hanitsch, Tessa Kerre, Robin Kobbe, Svetlana O. Sharapova, Kathrin Siepermann, Carsten Speckmann, Sophie Steiner, Nisha Verma, Jolan E. Walter, Emma Westermann-Clark, Sigune Goldacker, Klaus Warnatz, Markku Varjosalo, Bodo Grimbacher

**Affiliations:** ^1^ Institute for Immunodeficiency (IFI), Center for Chronic Immunodeficiency (CCI), Medical Center - University of Freiburg, Faculty of Medicine, University of Freiburg, Freiburg, Germany; ^2^ CIBSS – Centre for Integrative Biological Signalling Studies, Freiburg, Germany; ^3^ Institute of Biotechnology, University of Helsinki, Helsinki, Finland; ^4^ Faculty of Biology, University of Freiburg, Freiburg, Germany; ^5^ Department of Biosciences and Nutrition, NEO, Karolinska Institutet, Huddinge, Sweden; ^6^ Research Center for Immunodeficiencies, Children’s Medical Center, Tehran University of Medical Sciences, Tehran, Iran; ^7^ Clinical Immunology and Primary Immunodeficiencies Unit, Pediatric Allergy and Clinical Immunology Department, Hospital Sant Joan de Déu, Institut de Recerca Sant Joan de Déu, Barcelona, Spain; ^8^ Department of Surgery and Surgical Specializations, Facultat de Medicina i Ciències de la Salut, Barcelona, Spain; ^9^ RESIST – Cluster of Excellence 2155 to Hanover Medical School , Satellite Center Freiburg, Freiburg, Germany; ^10^ Department for Clinical Immunology and Rheumatology, Hannover Medical School, Hanover, Germany; ^11^ Department of Pediatrics, Division of Pediatric Hemato-Oncology and Stem Cell Transplantation, Ghent University Hospital, Ghent, Belgium; ^12^ Primary Immunodeficiency Research Lab, Center for Primary Immunodeficiency Ghent, Jeffrey Modell Diagnosis and Research Center, Ghent University Hospital, Ghent, Belgium; ^13^ Department of Immunology, Royal Free London NHS Foundation Trust, London, United Kingdom; ^14^ Institute of Immunity and Transplantation, University College London, London, United Kingdom; ^15^ Department of Pediatrics, Keck School of Medicine, University of Southern California and Children’s Hospital Los Angeles, Los Angeles, CA, United States; ^16^ HELIOS Children’s Hospital, Krefeld, Germany; ^17^ Laboratory of Clinical Immunology and Microbiology, National Institutes of Allergy and Infectious Diseases (NIAID), National Institutes of Health (NIH), Bethesda, MD, United States; ^18^ Department of Medical Immunology, Charité – Universitätsmedizin Berlin, Berlin, Germany; ^19^ Department of Hematology, Ghent University Hospital, Ghent, Belgium; ^20^ Institute for Infection Research and Vaccine Development (IIRVD), University Medical Center Hamburg-Eppendorf, Hamburg, Germany; ^21^ Research Department, Belarusian Research Center for Pediatric Oncology, Hematology and Immunology, Minsk, Belarus; ^22^ Center for Pediatrics and Adolescent Medicine, Medical Center - University of Freiburg, Faculty of Medicine, University of Freiburg, Freiburg, Germany; ^23^ Division of Allergy and Immunology, Department of Pediatrics, Morsani College of Medicine, University of South Florida, Tampa, FL, United States; ^24^ Division of Allergy/Immunology, Department of Pediatrics Johns Hopkins All Children’s Hospital, St. Petersburg, FL, United States; ^25^ Division of Allergy and Immunology, Massachusetts General Hospital for Children, Boston, MA, United States; ^26^ Division of Allergy and Immunology, Department of Medicine, Morsani College of Medicine, University of South Florida, Tampa, FL, United States; ^27^ Center for Chronic Immunodeficiency (CCI), Medical Center-University of Freiburg, Faculty of Medicine, University of Freiburg, Freiburg, Germany; ^28^ Department of Rheumatology and Clinical Immunology, Medical Center-University of Freiburg, Faculty of Medicine, University of Freiburg, Freiburg, Germany; ^29^ Helsinki Institute of Life Science, University of Helsinki, Helsinki, Finland; ^30^ Proteomics Unit, University of Helsinki, Helsinki, Finland; ^31^ DZIF – German Center for Infection Research, Satellite Center Freiburg, Freiburg, Germany

**Keywords:** primary immunodeficiency, inborn errors of immunity (IEI), NFKB1, common variable immunodeficiency (CVID), NF-kappaB signaling pathway

## Abstract

Most of the currently known heterozygous pathogenic *NFKB1* (Nuclear factor kappa B subunit 1) variants comprise deleterious defects such as severe truncations, internal deletions, and frameshift variants. Collectively, these represent the most frequent monogenic cause of common variable immunodeficiency (CVID) identified so far. *NFKB1* encodes the transcription factor precursor p105 which undergoes limited proteasomal processing of its C-terminal half to generate the mature NF-κB subunit p50. Whereas p105/p50 haploinsufficiency due to devastating genetic damages and protein loss is a well-known disease mechanism, the pathogenic significance of numerous *NFKB1* missense variants still remains uncertain and/or unexplored, due to the unavailability of accurate test procedures to confirm causality. In this study we functionally characterized 47 distinct missense variants residing within the N-terminal domains, thus affecting both proteins, the p105 precursor and the processed p50. Following transient overexpression of EGFP-fused mutant p105 and p50 in HEK293T cells, we used fluorescence microscopy, Western blotting, electrophoretic mobility shift assays (EMSA), and reporter assays to analyze their effects on subcellular localization, protein stability and precursor processing, DNA binding, and on the RelA-dependent target promoter activation, respectively. We found nine missense variants to cause harmful damage with intensified protein decay, while two variants left protein stability unaffected but caused a loss of the DNA-binding activity. Seven of the analyzed single amino acid changes caused ambiguous protein defects and four variants were associated with only minor adverse effects. For 25 variants, test results were indistinguishable from those of the wildtype controls, hence, their pathogenic impact remained elusive. In summary, we show that pathogenic missense variants affecting the Rel-homology domain may cause protein-decaying defects, thus resembling the disease-mechanisms of p105/p50 haploinsufficiency or may cause DNA-binding deficiency. However, rare variants (with a population frequency of less than 0.01%) with minor abnormalities or with neutral tests should still be considered as potentially pathogenic, until suitable tests have approved them being benign.

## Introduction

Heterozygous pathogenic sequence variants in *NFKB1* have been identified as the most frequent monogenic cause in common variable immunodeficiency (CVID)-like diseases (1–10, 11). *NFKB1* encodes the cytoplasmic transcription factor precursor p105, which undergoes limited proteasomal processing of its C-terminal half, whereby the shorter p50 subunit is generated (12–14). Both p105 and p50 are core molecules of the canonical NF-κB signaling pathway. Additional members of the NF-κB transcription factor family are the non-canonical p52 and its precursor p100 (encoded by *NFKB2*), RelA (also known as p65), RelB and cRel. All NF-κB proteins share the multifunctional N-terminal Rel-homology domain (RHD), which mediates interaction with inhibitor of NF-κB (IκB) proteins, dimerization, nuclear translocation and DNA-binding. The active NF-κB transcription factor complexes are composed of various homo- and hetero-dimeric combinations of the NF-κB and Rel proteins (15, 16). Only the Rel-proteins are equipped with a transactivation domain. Hence, assembling a transcriptional activator requires p50 (as well as p52) to interact with one of the Rel proteins, whereas p50 homodimers are repressors of transcription. The regulation of the NF-κB transcription factor activity is mainly achieved by retention of the dimers within the cytoplasmic compartment through IκB proteins (17).

Routine sequencing and systematic genetic studies in patients with suspected inborn errors of immunity (primary immunodeficiency diseases, PID) enabled the identification of numerous *NFKB1* sequence variants (7, 9–11). The pathogenic potential of these genetic lesions comprises “early” truncating mutations, predicting the expression of severely shortened, non-functional proteins and internal deletions, causing profound protein defects. In both cases, mRNA and/or protein decay is assumed to lead to insufficiency of p105 and p50. In fact, a recent comprehensive study has confirmed a deleterious character of most of the 41 investigated variants in this category (18). In addition, frameshift variants in the central part of p105, predict immediate expression of p50-like proteins, which by-pass the precursor stage (19, 20), as well as the sporadically occurring C-terminal truncations (21, 22), are also considered to have a pathogenic effect, although the disease-causing mechanisms are still unknown for both entities. The pathogenic significance of most (51/54 and 31/33) of the identified *NFKB1* missense alterations, however, remained uncertain (7, 10) unless experimental confirmation of their causality became available (18, 23). Amino acid changes localizing to the N-terminal half (aa 1-433) affect both the p105 precursor and the p50 subunit. In contrast, missense alterations in the C-terminal half of p105 (aa 434-969) are removed once (if) the precursor proteins undergo processing, which would convert them into wildtype p50 proteins (19).

In a recent report, we used a standard cell culture model, based on transient transfection of HEK293T cells with EGFP-fused p105 and p50 constructs, enabling functional assessments of *NFKB1* missense variants (23). Subsequent analyses, including expression and subcellular localization of the overexpressed proteins as well as p50-dependent DNA-binding and RelA-mediated reporter gene activation, confirmed a detrimental protein loss caused by a single amino acid change (p.Tyr350Cys) in a CVID family with p105/p50 haploinsufficiency.

In the current study, we assessed 47 distinct N-terminal p50 missense alterations, including 39 variants from the Tuijnenburg and Lorenzini cohorts, four variants described elsewhere (6–8, 10, 11, 18, 24–28) and four previously uncharacterized variants. We found nine single amino acid substitutions within the Rel-homology domain causing protein-decaying defects. Two variants caused a loss of the DNA-binding function, while protein stability remained unaffected. Several variants showed subtle abnormalities indicating diverse, yet unspecified functional impairment. We reconcile our findings with previous cohort studies (7, 10) and with a recent elegant functional *in vitro* report, in which p105/p50 deficient HEK293T cells were used in combination with a homodimerization-defective RelA mutant, to test 170 N-terminal missense variants for p50-dependent reporter gene activation 18). A set of case vignettes is attached and confirms the known variability of the *NFKB1* disease phenotypes.

## Materials and Methods

### *In silico* mutational analysis

The missense variants analyzed in this study have mostly been identified in previous studies (**Table 1** and References therein). The majority of the selected variants remained of uncertain significance (VUS), although their pathogenic potential has been estimated using *in silico* prediction tools. We restricted our functional characterization to missense variants residing with the N-terminal half of the p105 precursor, which corresponds to the mature p50, because our experimental tests (23) are not suitable for variants localizing to the processible C-terminal half (which generates wildtype p50 proteins in these cases). Each variant was manually assessed using commonly accessible platforms such as dbSNP (http://www.ncbi.nlm.nih.gov/snp); Genome Aggregation Database (https://gnomad.broadinstitute.org), Mutation Taster (www.mutationtaster.org), SIFT (https://sift.bii.a-star.edu.sg) Polyphen-2 (http://genetics.bwh.harvard.edu/pph2) and Ensembl Genome Browser (https://www.ensembl.org).

**Table 1:** Characteristics of the variants analyzed in this study.

cDNA change	Protein change (HGVS)	Protein change (single letter code)	pathogenic effect predictions (synopsis)	analyzed	p105 localization	p50 localization	p105 level and processing (Western Blot)	p50 level (Western Blot)	p105 transfection p50 DNA-binding (EMSA)	p50 transfection p50 DNA- binding (EMSA)	p105 level (Reporter assay)	Reporter activity p105	p50 level (Reporter assay)	Reporter activity p50	defect (synopsis)	assessment in Li et al.	Reference
c.16C>T	p.Pro6Ser	P6S	no	p50	nd	normal	nd	normal	nd	normal	nd	nd	normal	normal	no	―	Tuijnenburg
c.106G>A	p.Ala36Thr	A36T	no	p50	nd	normal	nd	normal	nd	normal	nd	nd	normal	normal	no	neutral	Lorenzini
c.115A>G	p.Thr39Ala	T39A	no	p50	nd	normal	nd	normal	nd	normal	nd	nd	normal	normal	no	neutral	Tuijnenburg; Li
c.131A>G	p.Tyr44Cys	Y44C	no	p50	nd	normal	nd	normal	nd	normal	nd	nd	normal	normal	no	―	Tuijnenburg
c.169C>T	p.Arg57Cys	R57C	yes	p105/p50	normal	normal	normal	reduced	absent	reduced	normal	reduced	reduced	reduced	yes	LOF	Lorenzini; Rojas-Restrepo
c.191G>T	p.Gly64Val	G64V	yes	p50	nd	normal	nd	normal	nd	reduced	nd	nd	normal	increased	yes	hypomorphic	Lorenzini
c.199C>T	p.His67Tyr	H67Y	weak	p50	nd	normal	nd	normal	nd	reduced?	nd	nd	normal	reduced	yes/unclear	hypomorphic	Lorenzini
c.200A>G	p.His67Arg	H67R	yes	p105/p50	normal	normal	normal	normal	reduced	reduced	normal	reduced	normal	normal	yes (**)	hypomorphic	Kaustio; Lorenzini
c.260T>G	p.Ile87Ser	I87S	yes	p105/p50	aberrant	aberrant	reduced	reduced	reduced	reduced	reduced	reduced	reduced	increased	yes	LOF	Tuijnenburg; Lorenzini
c.269A>C	p.Tyr90Ser	Y90S	yes	p105/p50	normal	normal	normal	reduced?	normal	normal	reduced?	normal	reduced	normal	yes/unclear	neutral	Lorenzini; Rojas-Restrepo
c.275G>T	p.Gly92Val	G92V	yes	p50	nd	aberrant	nd	reduced	nd	reduced	nd	nd	reduced	increased	yes	―	this study
c.293T>A	p.Val98Asp	V98D	yes	p105/p50	aberrant	aberrant	reduced	reduced	reduced	reduced	reduced	reduced	reduced	reduced?	yes	LOF	Tuijnenburg; Lorenzini
c.307A>G	p.Asn103Asp	N103D	no	p105/p50	normal	normal	normal	normal	reduced	normal	normal	normal	normal	increased	no/unclear	―	this study
c.406G>A	p.Gly136Ser	G136S	no	p105/p50	normal	normal	normal	normal	normal	normal	normal	normal	normal	increased	no	neutral	Tuijnenburg; Li; this study;
c.419T>A	p.Leu140Gln	L140Q	yes	p50	nd	normal	nd	normal	nd	normal	nd	nd	normal	increased?	no	―	Govindarajan
c.425T>C	p.Ile142Thr	I142T	yes	p50	nd	aberrant	nd	reduced	nd	reduced	nd	nd	reduced	increased	yes	LOF	Duan
c.470G>C	p.Arg157Pro	R157P	yes	p105/p50	aberrant	aberrant	reduced	reduced	reduced	reduced	reduced	reduced	reduced	increased	yes	LOF	Schröder; Lorenzini
c.556G>T	p.Asp186Tyr	D186Y	weak	p50	nd	normal	nd	normal	nd	normal	nd	nd	normal	normal	no	neutral	Lorenzini
c.574C>T	p.Arg192Trp	R192W	weak	p50	nd	normal	nd	normal	nd	size?	nd	nd	normal	normal	no/unclear	―	Tuijnenburg
c.586C>G	p.Leu196Val	L196V	no	p50	nd	normal	nd	normal	nd	normal	nd	nd	normal	normal	no	―	this study
c.592C>T	p.Arg198Cys	R198C	weak	p105/p50	normal	normal	normal	normal	normal	normal	normal	normal	normal	increased?	no/unclear	neutral	Lorenzini
c.593G>A	p.Arg198His	R198H	no	p50	nd	normal	nd	normal	nd	normal	nd	nd	normal	normal	no	neutral	Tuijnenburg; Li
c.604C>A	p.Leu202Met	L202M	no	p50	nd	normal	nd	normal	nd	normal	nd	nd	normal	normal	no	―	Tuijnenburg
c.641G>A	p.Arg214Gln	R214Q	yes	p105/p50	normal	normal	normal	reduced?	normal	normal	normal	reduced?	reduced	normal	yes/unclear	neutral	Lorenzini; Rojas-Restrepo
c.646A>G	p.Met216Val	M216V	yes	p105/p50	normal	normal	normal	normal	normal	normal	normal	reduced?	reduced	reduced	yes/unclear	neutral	Lorenzini; Rojas-Restrepo
c.689G>A	p.Arg230Lys	R230K	weak	p105/p50	normal	normal	normal	normal	normal	normal	normal	normal	normal	normal	no	neutral	Lorenzini
c.691C>T	p.Arg231Cys	R231C	weak	p50	nd	normal	nd	normal	nd	normal	nd	nd	normal	normal	no	neutral	Anim
c.734C>T	p.Ala245Val	A245V	yes	p105/p50	normal	normal	normal	normal	reduced	reduced?	normal	normal	normal	increased	yes/unclear	neutral	Lorenzini
c.736C>A	p.Pro246Thr	P246T	yes	p105/p50	normal	normal	normal	normal	reduced	normal	normal	normal	normal	normal	no	neutral	Lorenzini
c.843C>G	p.Ile281Met	I281M	yes	p105/p50	normal	normal	normal	normal	reduced	normal	normal	reduced?	reduced?	increased	yes/unclear	neutral	Tuijnenburg; Lorenzini
c.851G>C	p.Arg284Pro	R284P	yes	p50	nd	aberrant	nd	reduced	nd	reduced	nd	nd	reduced	increased	yes	―	this study
c.856T>A	p.Tyr286Asn	Y286N	yes	p105/p50	aberrant	aberrant	reduced	reduced	reduced	reduced	reduced	reduced	reduced	increased	yes	hypomorphic	Lorenzini
c.885G>C	p.Trp295Cys	W295C	yes	p105/p50	aberrant	aberrant	reduced	reduced	reduced	reduced	reduced	reduced	reduced	increased	yes	LOF	Lorenzini
c.965T>C	p.Ile322Thr	I322T	no	p50	nd	normal	nd	normal	nd	normal	nd	nd	normal	normal	no	neutral	Tuijnenburg
c.967A>T	p.Asn323Tyr	N323Y	weak	p50	nd	normal	nd	normal	nd	normal	nd	nd	normal	normal	no	―	Christiansen
c.978A>C	p.Lys326Asn	K326N	weak	p50	nd	normal	nd	normal	nd	normal	nd	nd	normal	normal	no	neutral	Lorenzini; Li
c.1004G>A	p.Arg335Gln	R335Q	weak	p50	nd	normal	nd	normal	nd	normal	nd	nd	normal	normal	no	neutral	Lorenzini
c.1049A>G	p.Tyr350Cys	Y350C	yes	p105/p50	aberrant	aberrant	reduced	reduced	reduced	reduced	reduced	reduced	reduced	increased	yes	LOF	Lorenzini; Fliegauf
c.1115C>T	p.Ser372Leu	S372L	no	p105/p50	normal	normal	normal	normal	reduced	normal	normal	normal	normal	normal	no	neutral	Lorenzini
c.1126G>A	p.Gly376Ser	G376S	no	p105/p50	normal	normal	normal	normal	normal	normal	normal	normal	normal	normal	no	neutral	Lorenzini
c.1129G>A	p.Gly377Ser	G377S	no	p50	nd	normal	nd	normal	nd	normal	nd	nd	normal	normal	no	neutral	Tuijnenburg; Li
c.1147G>T	p.Ala383Ser	A383S	no	p105/p50	normal	normal	normal	normal	normal	normal	normal	normal	normal	normal	no	neutral	Lorenzini
c.1156G>A	p.Gly386Arg	G386R	weak	p105/p50	normal	normal	normal	normal	normal	normal	normal	normal	normal	normal	no/unclear (*)	neutral	Tuijnenburg; Lorenzini; Li
c.1177G>A	p.Gly393Ser	G393S	no	p50	nd	normal	nd	normal	nd	normal	nd	nd	normal	reduced?	no	neutral	Lorenzini
c.1214A>G	p.Tyr405Cys	Y405C	no	p50	nd	normal	nd	normal	nd	normal	nd	nd	normal	increased	no	neutral	this study
c.1226A>T	p.His409Leu	H409L	no	p50	nd	normal	nd	normal	nd	normal	nd	nd	normal	normal	no	neutral	Tuijnenburg
c.1289G>A	p.Gly430Glu	G430E	weak	p50	nd	normal	nd	normal	nd	normal	nd	nd	normal	normal	no	―	Yang

(*) weak evidence only observed in pre-experiments (data not shown); (**) this variant has previously been described as pathogenic. LOF, loss-of-function; nd, not determined.

### Cell culture and transfection

All *in vitro* methods used in this study were previously described in extensive detail (1, 23). Briefly, HEK293T (human embryonic kidney) cells were grown in Dulbecco`s Modified Eagle Medium (DMEM) supplemented with 10% fetal bovine serum (FBS) and 1% penicillin/streptomycin under standard conditions and plated onto collagen-coated 48-well culture plates or glass coverslips prior to transfection with plasmid DNA constructs using X-tremeGENE HP reagent (Roche, Mannheim, Germany). Mutations were introduced into the cDNAs encoding N-terminally EGFP-tagged wildtype p105 or p50 by site-directed mutagenesis using overlap-extension PCR.

### Fluorescence microscopy

Cells were seeded onto collagenized glass coverslips in 24-well plates and transfected with the indicated constructs. Cells were fixed with 4% paraformaldehyde (PFA), nuclei were stained with Hoechst 33342 (Sigma/Merck, Darmstadt Germany) and coverslips were mounted onto glass slides. Microscopic photographs were taken using a Zeiss Axio Observer (Carl Zeiss, Jena, Germany) equipped with a 40x/0.75 objective and processed with ZEN-blue software.

### Western blot analysis

Crude cell lysates were prepared in radio-immunoprecipitation assay buffer (RIPA) buffer (50 mM Tris pH 8, 1% Igepal, 0.5% sodium-deoxycholate, 150 mM NaCl, 1 mM ethylenediaminetetraacetic acid (EDTA), 0.1% sodium dodecyl-sulfate (SDS), 1% protease inhibitor cocktail) and separated on discontinuous 5%/9% Bis-Tris polyacrylamide gels, transferred to polyvinylidene difluoride (PVDF) membranes and processed for detection with an Odyssey CLx infrared scanner (LI-COR Biosciences, Bad Homburg, Germany). Primary antibodies were rabbit-anti-NF-κB1 #13586 (raised against residues surrounding Ile415 of mouse NF-κB1 to simultaneously detect p105 and p50) and mouse-anti-beta-actin #3700 (both from Cell Signaling; NEB; Frankfurt, Germany). Signals were detected with IRDye-coupled goat-anti-rabbit and goat-anti-mouse secondary antibodies (LI-COR).

### Electrophoretic mobility shift assay

Nuclear proteins were prepared from transfected cells following previously described procedures (29) using a modified buffer A (10 mM HEPES-KOH pH 7.9, 1.5 mM MgCl_2_, 0.1 mM EDTA, 10 mM KCl, 0.05% NP40, 0.5 mM dithiothreitol (DTT), 1% protease inhibitor cocktail). Binding reactions with DY681-labelled annealed oligos (forward 5`– AGT TGA GGG GAC TTT CCC AGG C – 3` and reverse 5`- GCC TGG GAA AGT CCC CTC AAC T -3`) were carried out in 1x binding buffer (10mM Tris pH 7.4; 1mM EDTA; 100mM KCl; 0.25mM DTT; 0.25% Tween-20; 5% glycerol; 0.01% BSA; 100ng/µl poly-dI:dC). Samples were separated on 6% polyacrylamide/1x Tris-acetate-EDTA (TAE) gels and visualized with an Odyssey CLx infrared scanner (LI-COR).

### Fluorescence-based reporter assay

An NF-κB responsive red-fluorescence reporter was generated by replacing the CMV-promoter in the ptdTomato vector (Takara/Clontech; Saint-Germain-en-Laye, France) with an artificial promoter composed of five NF-κB consensus binding sites, introduced *via* synthetic oligonucleotides at the 5`end of a cytomegalovirus (CMV) minimal promoter fragment. Expression vector constructs for N-terminally EGFP-tagged wildtype or mutant p105 or p50 (300ng per well each) and non-tagged RelA (5ng per well if not indicated otherwise) were transfected together with the reporter gene vector (100ng per well) into HEK293T cells grown on collagenized 48-well plates. Non-transfected, reporter-only and reporter-plus-RelA-only samples were included as controls. Fluorescence intensities were determined in live cells using a FluoroSpot Analyzer (CTL Immunospot, Bonn, Germany) with separate recordings of the tdTomato (red) and the EGFP (green) signals in each well. Plates were scanned repeatedly within 42-48 and 66-72 hours after transfection using variable magnifications and exposure settings. Fluorescence values were quantified with ImageJ (30) and normalized to the “reporter only” baseline control which was defined as 1-fold.

### Generation of stable cell lines with inducible NFKB1 expression and mass spectrometry

NFKB1 constructs, cloned with N-terminal MAC-tags, were used to generate stable cell lines from Flp-In T-REx 293 cell lines (Invitrogen, Life Technologies, R78007). Cell lines construct expression was induced, at 70% confluency, with 2mg/ml tetracycline and for BioID samples, additional 50mM biotin was added. Cells were harvested 24 hours after induction. Two biological replicates were prepared for both affinity purification coupled with mass spectrometry (AP-MS) and BioID, for each cell line, with 1.5*10^8 cells harvested per sample. Interaction analysis was done according to the previously described workflow (31, 32). Each sample was analyzed twice as technical replicates.

## Results

### Missense variants residing within the Rel-homology domain of p50 frequently gain high *in silico* effect prediction scores, indicating deleterious protein defects

**Figure 1:**
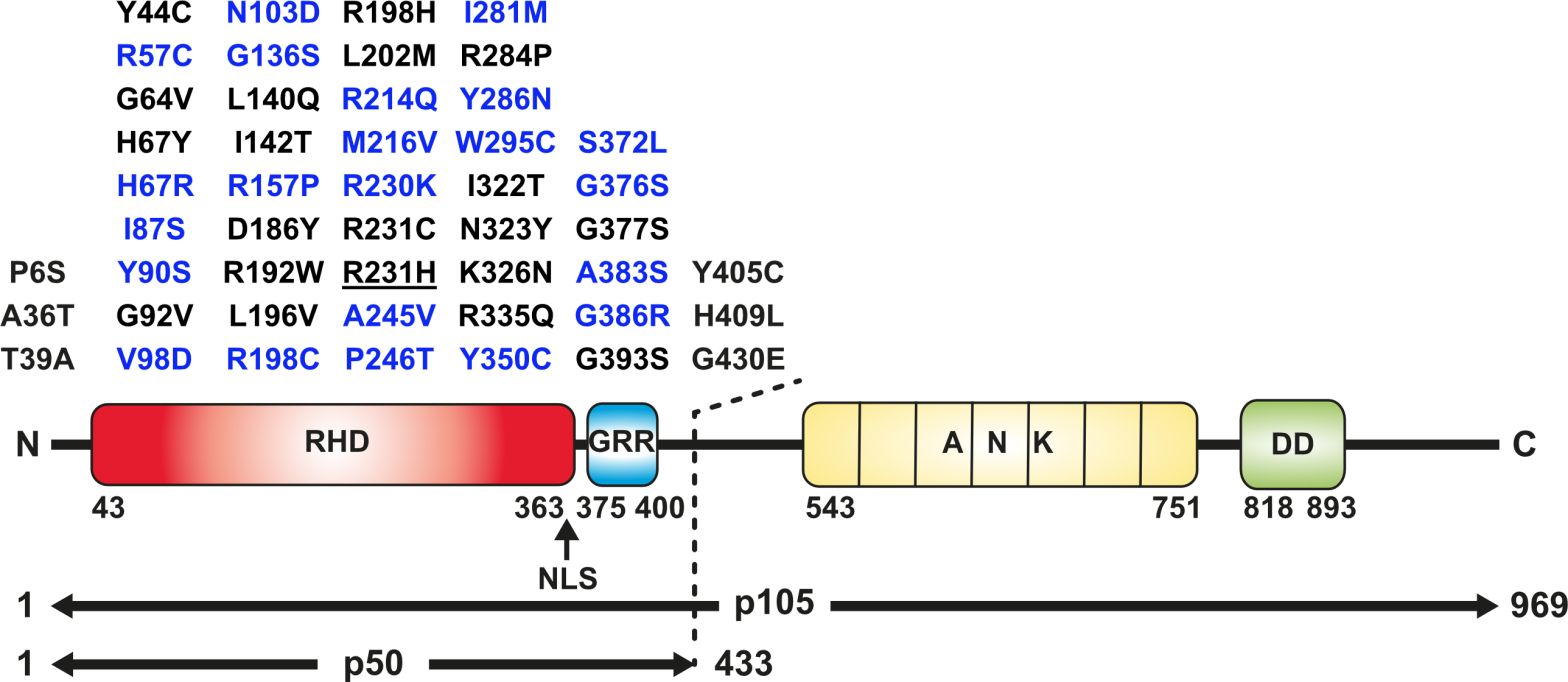
Missense variants analyzed in this study and domain structure of p105/p50 (Upper panel) Amino acid changes localizing to the N-terminal half of p105 affect both the precursor and the mature p50. Blue, variants tested in p105 and p50; black, variants tested in p50 only. The panel comprises all p50 variants enrolled in the Tuijnenburg and Lorenzini studies, except R231H (underlined). The deleterious variant Y350C has previously been described (23) and was included as a prototypical control. (Lower panel) The protein domain structure of the p105 precursor (long horizontal arrow) with the Rel-homology domain (RHD; red), glycine-rich region (GRR; blue), Ankyrin-repeat domain (ANK; yellow) and death domain (DD; green). Removal of the C-terminal half by limited proteolysis generates the mature transcription factor subunit p50 (short horizontal arrow). Numbers denominate amino acid positions. The position of the nuclear localization sequence (NLS) is indicated by an arrow.

In this study, we analyzed 47 *NFKB1* missense variants for deleterious effects. All variants reside within the N-terminal half of the p105 precursor protein, which corresponds to the mature p50 (**Figure 1**). We employed a previously introduced *in vitro* procedure, comprising transient overexpression of N-terminally tagged EGFP fusion constructs of mutant p105 and/or p50 proteins in HEK293T cells. The ectopically expressed proteins were subsequently analyzed for stability and sustainability, for their sub-cellular localization and DNA-binding activity and for their ability to activate a reporter gene (23). For a full-panel analysis including both p105 and p50 we selected 22 variants, which were either identified in our cohort or were previously confirmed or assumed to exert pathogenic effects (**Table 1**, **Supplementary Table 1** and references therein). Additional 25 variants were included in functional analyses of the p50 subunit only. These were either obtained from the literature or identified in patients from collaborating institutions (**Figure 1**).

All 47 missense variants were manually subjected to *in silico* analyses using various databases and effect prediction tools (**Supplementary Table 1**). A total of 19 variants were consistently scored with high pathogenicity values, while 11 variants had variable but weak predictions and 17 had low scores or were classified to be benign. Of note, the effect predictions in the ENSEMBL genome browser, suggest the presence of two hot-spot subdomains within the Rel-homology domain of p50 gaining generally elevated scores (aa 40-90 and aa 235-310, respectively). All *NFKB1* sequence variants listed in the ENSEMBL and gnomAD databases are heterozygous, although frequent variants with low prediction scores might sporadically occur in homozygosity, if protein function is not impaired.

### Deleterious missense variants cause accelerated decay of cytoplasmic p105 and sub-nuclear disposal of p50

**Figure 2:**
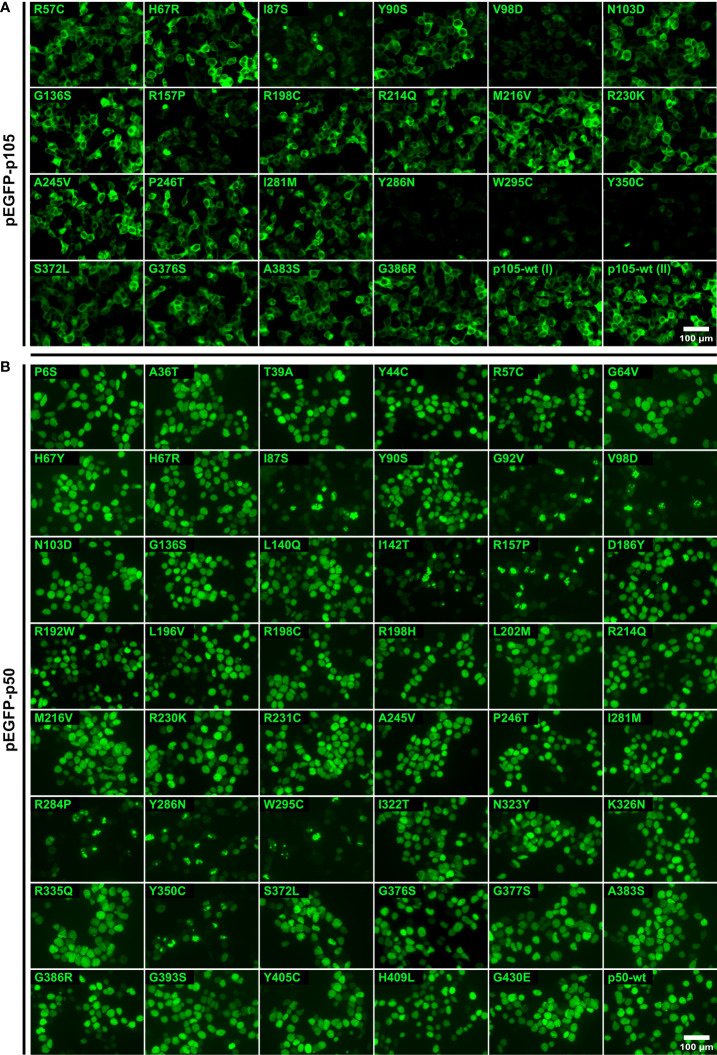
Deleterious missense variants causing protein loss are characterized by weak p105 expression and sub-nuclear deposition of p50. HEK293T cells were transiently transfected with expression vectors encoding EGFP-tagged proteins of wildtype or mutant p105 or p50 as indicated and analyzed by fluorescence microscopy. The known devastating Y350C variant was included as a control. Scale bars are indicated. Overlay images with stained nuclei are shown in Supplementary Figures 1A, B. Representative results are shown. **(A)** Ectopically expressed p105 predominantly localizes to the cytoplasm. EGFP-fused wildtype p105 and non-decaying variants yield robust expression levels. Limited expression and/or aberrant localization indicate severe protein defects (I87S, V98D, R157P, Y286N, W295C, and Y350C). **(B)** Transiently overexpressed EGFP-tagged wildtype p50 and non-decaying variants show a homogeneous nuclear distribution. Deleterious protein defects are indicated by unusual sub-nuclear protein deposition into aggregate-like structures with high fluorescence intensities (I87S, G92V, V98D, I142T, R157P, R284P, Y286N, W295C and Y350C).

Following overexpression of the EGFP-fused p105 proteins in HEK293T cells, conventional epi-fluorescence microscopy showed robust protein levels and almost exclusive cytoplasmic localization of the wildtype p105 and most of the analyzed variants (**Figure 2A**). In contrast, clearly overall reduced signal intensities and signal accumulation in highly intense spots were observed for the N-terminally located variants I87S, V98D and R157P. A strong reduction of EGFP-signals was obtained with three variants (Y286N, W295C, and the previously described Y350C) localizing to the C-terminal part of the Rel-homology domain. Quantification of the signal intensities in live cells using a Fluorospot reader, confirmed the microscopic observations (see below). As described previously (1, 8, 23) severely reduced signal intensities upon forced expression of mutant p105 proteins indicate accelerated decay due to deleterious protein defects and predicts a pathogenic effect of the underlying genetic variant.

Although under physiological conditions the nuclear translocation of dimeric NF-κB transcription factors depends on the degradation of the inhibitory cytoplasmic component (IκB), overexpressed EGFP-p50 shows a strictly nuclear localization with a homogeneous distribution *in vitro* (1, 8, 23). In contrast, pathogenic variants causing deleterious p50 defects and accelerated protein decay, only gain limited expression levels and show a typical sub-nuclear accumulation of the fluorescence signals in high intense spot-like structures. The characteristic aberrant pattern with pathogenic sub-nuclear protein disposal was observed in nine of the 47 analyzed variants (**Figure 2B**). These again included N-terminally located variants (I87S, G92V, V98D, I142T, and R157P), and variants affecting the C-terminal region of the Rel-homology domain (R284P, Y286N, W295C and Y350C). All other variants had a uniform nuclear distribution indistinguishable from the overexpressed wildtype p50. These observations were confirmed by quantitative measurements of the signal intensities in automated microscopic fluorescence recordings (see below). Interestingly, although no devastating protein loss was detectable, several variants including R57C, Y90S and I281M, showed diminished fluorescence levels, suggesting subtle or less severe protein defects.

### Severe missense defects destabilize p105, prevent precursor processing and render p50 proteins unsustainable

**Figure 3:**
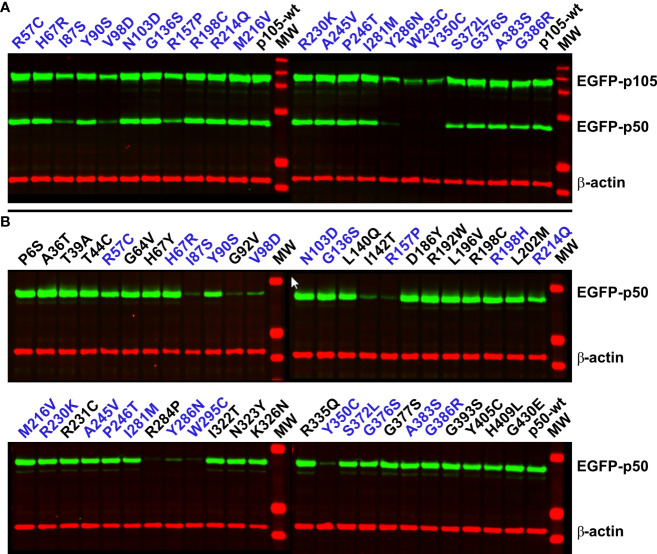
Damaging NFKB1 variants cause rapid p105 decay, abrogate processing of p105 to generate p50, and prohibit a sustained abundance of p50. HEK293T cells were transiently transfected with EGFP-fusion constructs either encoding p105 or p50 missense variants as indicated. Variants depicted in blue font were included in both panels. Whole-cell lysates were analyzed by Western blotting using antibodies directed against an epitope near the C-terminal end of p50, to simultaneously detect p105 and p50 (both green), and against β-actin (red) as loading control. **(A)** In cells transfected with wildtype p105 or non-decaying p105 mutants, an invariant proportion of the ectopically expressed p105 is converted to p50 by endogenous mechanisms. Deleterious variants are identified by weaker p105 expression and low or undetectable p50 (I87S, V98D, R157P, Y286N, W295C, and Y350C). Representative results of five independent experiments are shown. **(B)** Upon enforced expression of p50 (skipping the precursor stage) deleterious variants only gain limited expression levels (I87S, G92V, V98D, I142T, R157P, R284P, Y286N, W295C and Y350C). Representative results of six independent experiments are shown. Samples were blotted once in two experiments and at least twice in four experiments.

In cells overexpressing p105, an invariable amount of the ectopic precursor protein is converted into p50 *via* removal of its C-terminal half by endogenous mechanisms (23). Therefore, we tested next, whether p105 expression and p50 processing is affected in the mutant precursor proteins (**Figure 3A**). All 22 variants for which we generated p105 expression constructs were transfected into HEK293T cells and analyzed by Western blotting. For the variants I87S, V98D and R157P we observed both, markedly reduced p105 expression and diminished p50. The effects were even more pronounced when the p105 variants Y286N, W295C and Y350C were transfected. As previously described, immediate elimination of mutant p105 precursor proteins and inefficient or disabled conversion into (non-sustainable) p50 indicates deleterious missense defects (23). All other variants were indistinguishable from the wildtype control.

Western blotting of the complete set of 47 variants for which we generated p50 expression constructs, yielded a single protein band in each case (**Figure 3B**). Yet, only weak or very faint bands were obtained with nine of the variants (I87S, G92V, V98D, I142T, R157P, R284P, Y286N, W295C and the previously described Y350C). These results are consistent with the microscopic analyses and indicate the weak abundance of these proteins due to severe protein defects. In contrast to the clearly deleterious mutants, several variants presented with only mildly reduced p50 levels, including R57C, Y90S, R214Q and I281M. Here, less severe defects might be associated with moderately accelerated protein decay.

### Identification of two non-decaying *NFKB1* missense variants causing loss of DNA-binding function

**Figure 4:**
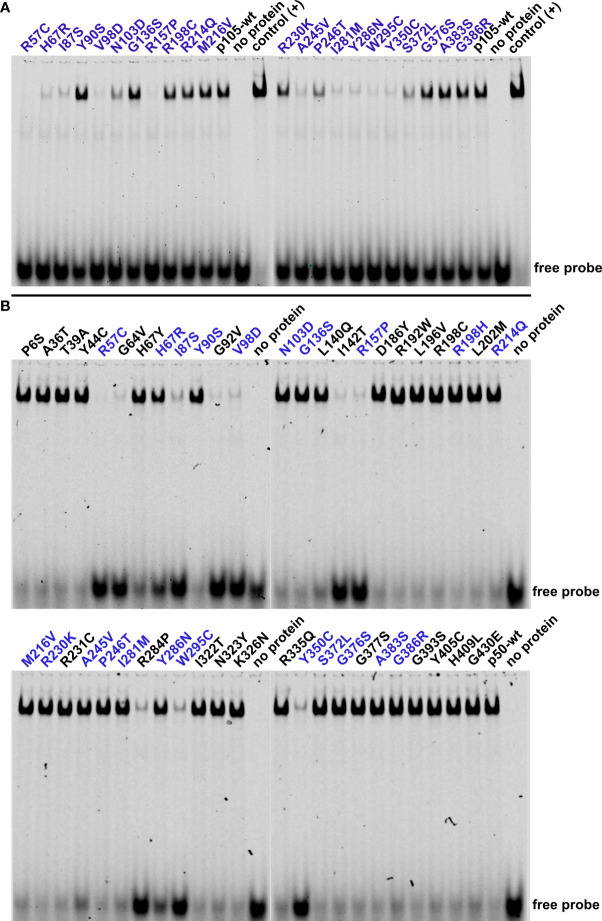
Impaired p50-mediated DNA-binding activity uncovers deleterious NFKB1 mutations and loss-of-function variants. HEK293T cells were transiently transfected with the indicated p105 or p50 variants. NF-κB DNA-binding activities were determined by EMSA using nuclear extracts. **(A)** In cells transfected with p105 expression constructs, nuclear DNA-binding activity originates from cell-intrinsic generation of p50 by processing of precursor proteins. Reduced or absent DNA-binding denotes decaying and DNA-binding deficient variants or might indicate subtle defects e.g. related to processing or nuclear transfer. Please note the consumption of the free probe when using overexpressed p50 as positive control. Representative results of three independent experiments are shown. Electrophoresis was carried out twice. **(B)** Immediate expression of p50 (i.e. not generated *via* precursor processing) indicates deleterious p50 defects and DNA-binding-deficiency. Please note the reduced size of the shifted band with the R192W variant. Representative results of five independent experiments are shown.

DNA-binding of NF-κB transcription factors is mediated by the Rel-homology domain. Since 36 of the 47 *NFKB1* variants tested in this study localize to the Rel-homology domain, we next used electrophoretic mobility shift assays (EMSA) to assess the DNA-binding activity of the mutant p50 proteins (**Figure 4**). Following transfection of p105, partial conversion of the ectopic protein into p50 by cell-intrinsic mechanisms yields a detectable DNA-binding activity, which however is considerably weaker compared to directly overexpressed p50 (23). Upon transfection of all 22 precursor variants, only nine were indistinguishable from the p105 wildtype control, indicating normal processing, nuclear translocation and DNA-binding (**Figure 4A**). In contrast, all six deleterious p105 variants (I87S, V98D, R157P, Y286N, W295C and Y350C) produced almost undetectable or only weak DNA-binding, compatible with intensified decay and protein loss, as expected. However, seven normally expressed variants had undetectable or only weak (R57C, H67R, A245V and I281M) or mildly reduced (N103D, P246T and S372L) DNA-binding activities, suggesting the presence of non-decaying protein defects.

We then assessed the DNA-binding activities following transfection of the 47 mutant p50 constructs, aiming at immediate overexpression of nuclear p50 proteins, while skipping the precursor stage (**Figure 4B**). Among the nine deleterious variants within the p50 panel, eight showed only weak DNA-binding activity (I87S, G92V, V98D, I142T, R157P, R284P, W295C and Y350C), compatible with protein loss (rather than loss of DNA-binding activity *per se*). For the p50-Y286N variant, binding to DNA was reduced (indicated by an excess of the free probe), but clearly detectable. Remarkably, DNA-binding was almost absent in the non-decaying variants R57C and G64V, suggesting a pathogenic mechanism, which is not based on protein loss as observed in NF-κB1 insufficiency. Therefore, R57C and G64V represent loss-of-function missense variants (in the strict sense, i.e. loss-of-activity as opposed to loss-of-expression), characterized by normal stability of both p105 and p50, but absent DNA-binding ability of the mutant p50. DNA-binding was moderately diminished in the normally expressed H67Y, H67R and A245V variants and marginally altered (if at all) with the L140Q, R214Q, M216V and I281M variants. DNA-binding was normal in three p50 variants (N103D, P246T, S372L) but reduced upon overexpression of their mutant p105 precursor proteins, suggesting a functional defect that does not originate from impaired protein stability or DNA-binding.

In summary, the combination of expression analyses and EMSA can reliably detect both protein-decaying variants and DNA-binding defects, particularly upon direct expression of the nuclear p50 subunits. However, subtle protein defects such as impaired IκB-interaction, disturbed nuclear translocation or disabled transcription factor dimerization, remain unidentified.

### Missense variants of nuclear p50 can alter the ability of NF-κB dimers to regulate reporter gene transcription

**Figure 5:**
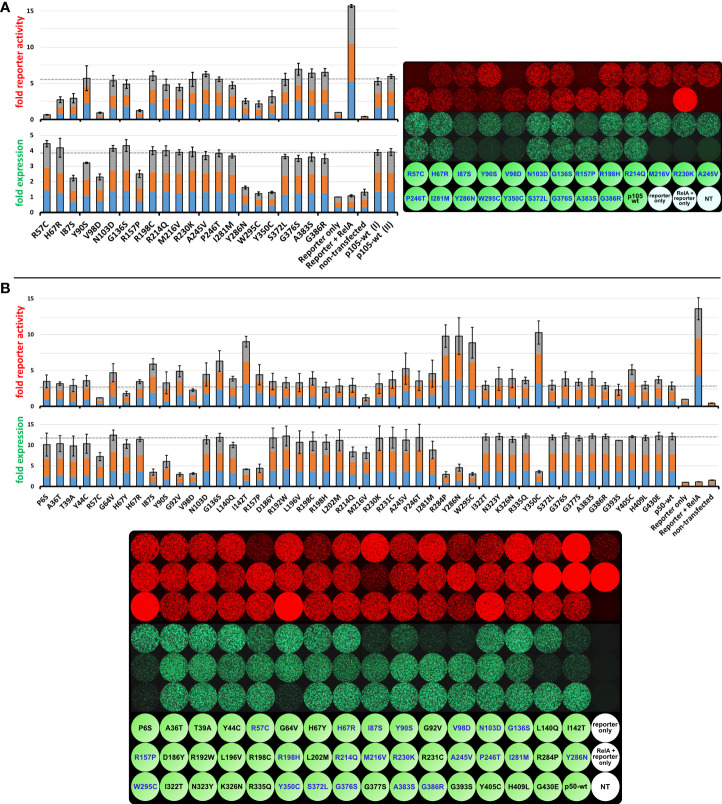
The inhibitory effect on RelA-dependent promoter activation can be augmented by co-expression of mutant p105 but can either be attenuated or intensified by mutant p50. HEK293T cells were transiently transfected with a synthetic reporter gene construct (100ng), composed of an NF-κB responsive promoter driving the expression of the red fluorescent protein tdTomato. Reporter expression was switched on to maximum levels by co-expression of RelA (5 to 7.5ng vector). An excess of wildtype or mutant EGFP-p105 or EGFP-p50 expression vectors (300ng each; green fluorescence) was added to inhibit the RelA-mediated reporter activation. Expression of the reporter gene and the p105- or p50 fusion constructs was monitored at 48h (not shown) and 72h after transfections by recording the red (4-8 scans per plate) and green (3-6 scans per plate) fluorescence intensities, respectively. Average fold values were calculated from three independent experiments with the baseline intensities of the “reporter only” controls set as 1-fold. Each color in the three-colored bar graphs indicates one experiment (proportional illustration, no absolute values). Microscopic images show one representative example of the three independent experiments each. The full panels with absolute reporter activity and expression values for each variant are shown in the Supplementary Figures 2-3 and Supplementary Table 2. **(A)** Moderate RelA-dependent reporter activity is observed with co-expression of wildtype p105. Decreased reporter expression upon co-expression of mutant p105 compared to wildtype p105 indicates protein defects and might be due to less or absent processing to p50 and/or cytoplasmic retention of RelA by mutant p105, respectively. Average values were calculated from three independent experiments using 5ng, 6ng and 7.5ng of the RelA vector, respectively. **(B)** Co-expression of wildtype p50 limits the RelA-dependent reporter activation, probably due to the assembly of excess homodimeric transcriptional repressors. Defects of p50 caused by single amino acid changes are indicated by either reduced or increased reporter activation compared to wildtype p50 and might be due to less expression, loss of DNA binding activity or other defects. Average values were calculated from three independent experiments using 5ng RelA vector each.

To test whether *NFKB1* missense variants can interfere with the RelA-mediated transcriptional activation, we used a reporter competition assay in which an NF-κB-responsive promoter drives the expression of a red-fluorescent protein (23). The assay is based on the p105- and/or p50-mediated inhibition of the RelA-dependent reporter activation and requires co-transfection of a RelA expression vector (to switch on the reporter) together with EGFP-fusion constructs of either p105 or p50 in addition to the synthetic reporter gene into HEK293T cells. In subsequent intervals, both the p105/p50 expression (green) and the reporter activity (red) were monitored in live cell cultures by fluorescence intensity measurements using automated microscopic imaging (**Figure 5**; **Supplementary Figure 2, 3**; **Supplementary Table 2**).

Upon transfection of the 22 constructs encoding the EGFP-fused p105 variants, expression of all six deleterious variants was limited (green fluorescence), as expected (**Figure 5A**; **Supplementary Figure 2**). Moderately reduced EGFP signals were observed with the N-terminal variants (I87S, V98D, R157P), whereas only low protein levels were obtained when the missense changes resided further downstream, within the C-terminal part of the Rel-homology domain (Y286N, W295C, Y350C). In addition, the Y90S variant caused a mild reduction whereas expression of all other variants was comparable to the wildtype control (**Figure 5A** bar graphs).

Co-transfection of RelA alone together with the reporter vector gained strong reporter signals (red fluorescence), most likely due to assembly of highly potent homodimeric transcriptional activators (**Figure 5A**). Upon co-transfection of wildtype or mutant p105 together with RelA and the reporter gene, RelA-mediated reporter activation can, in principle, be attenuated *via* two mechanisms. First, by means of the IκB-like activity mediated by the C-terminal half of the overexpressed cytoplasmic p105 precursor proteins and second, by its proteasomal processing products p50 which lacks a transactivation domain. The latter enables assembly of less potent p50:RelA heterodimeric transcriptional activators as well as p50:p50 homodimeric repressors. Of note, all missense changes analyzed in the current study reside within the N-terminal p50 half, whereas the IκB-like C-terminal half is wildtype in each case. In addition to the mere presence of p105 and p50, reporter competition might be variably modulated by loss-of-DNA-binding function and other undefined factors such as impaired protein-interactions or nuclear translocation.

Among the deleterious p105 variants only V98D and R157P gained a strong repressive effect, whereas the RelA-mediated reporter activation (red fluorescence) was moderately repressed by the I87S and the Y286N, W295C and Y350C variants, respectively (**Figure 5A**). Remarkably, the non-decaying but DNA-binding-deficient R57C variant almost completely blocked reporter activation (G64V has not been tested in the p105 panel). Among the p105 variants, which produced only limited DNA-binding activities when transfected alone (H67R, N103D, A245V, P246T, I281M and S372L; as described above), only H67R showed reduced reporter activation, whereas all others had reporter activities comparable to the overexpressed wildtype p105. These observations suggest, that the main inhibitory effect on RelA-dependent reporter activation in our assay is promoted by the IκB-like activities of the overexpressed p105 variants (mediated by the non-mutant C-terminal half), rather than by mutant p50 proteins.

Since the generation of p50 from ectopic p105 is dependent on cell-intrinsic processes, and reporter activation likely includes a complex involvement of endogenous NF-κB proteins, the reliability of these functional *in vitro* analyses is basically limited to the deleterious variants. The decaying variants can be easily discriminated by the simultaneous monitoring of the expression levels, whereas hypomorphic defects require more detailed analyses. Therefore, to analyze the direct, precursor-independent effect of the *NFKB1* missense variants on the transcriptional activation/repression function of nuclear p50, we subjected the complete panel of our p50 expression constructs to the reporter competition test (**Figure 5B**, **Supplementary Figure 3**; **Supplementary Table 2**).

Expression levels of the EGFP-fused p50 proteins were determined by automated signal readings (green fluorescence) and were consistent with Western blotting results. Five of the nine protein-decaying variants (I142T, R284P, Y286N, W295C and Y350C) showed weakly impaired RelA-mediated reporter activation (red fluorescence), consistent with profound protein loss. Three of the deleterious variants (I87S, G92V and R157P) gained reporter activities, which were moderately above the p50 wildtype control levels, whereas the V98D variant was clearly below. Therefore, although devastating variants commonly lead to accelerated elimination of the mutant proteins, their enforced nuclear presence can have distinct effects on RelA-dependent reporter gene activation, e.g. when sub-nuclear disposal of mutant p50 aggregates consumes variable amounts of RelA (23). Interestingly, the two non-decaying but DNA-binding deficient variants had opposing effects, with complete loss of reporter activity (R57C) and marginally increased signal intensities (G64V) compared to the wildtype control. It appears possible that p50:p50 homodimers are differently affected by the loss of the DNA-binding function of both subunits (as tested by EMSA) than the heterodimeric p50:RelA transcription factors (as tested by reporter assays), which however has not been further pursued in this study. Reporter activity was also differently affected by the H67Y and H67R variants, with a clear reduction only with p50-H67Y, although both had comparable expression and DNA-binding activities.

Although G136S and Y405C were neutral in expression and DNA-binding analyses, both gained clearly increased reporter activities compared to the control, whereas reporter activity was only at baseline levels with the otherwise neutral M216V variant. Three variants (N103D, A245V and I281M), all of which had impaired DNA-binding activity when expressed as precursor mutants but which otherwise were normal (except for a slightly diminished expression of the p50-I281M), caused increased reporter activities compared to the wildtype control. This effect was most prominent with the A245V variant. Several variants had minor effects on the reporter activity such as the G393S variant (slightly reduced) and the R198C and R231C variants (both slightly increased), which we, however, did not consider as relevant defects.

In summary, our reporter assay with mutant p105 proteins primarily indicates deleterious variants by their RelA-mediated promoter activation below the p105 wildtype levels. In contrast, reporter assays with mutant p50 proteins can either yield increased or decreased signal intensities compared to the wildtype control. In both cases, the decaying variants are easily identified by their expression levels, whereas normal protein stability plus aberrant reporter activity indicates functional defects other than protein loss. Among these, only one of the two DNA-binding deficient variants was trackable. Subtle functional defects such as disturbed precursor processing, altered protein-protein interactions or impaired nuclear translocation cannot be further specified using transient overexpression systems. We therefore assume that many non-decaying pathogenic p105/p50 defects will remain unidentified, unless appropriate tests are conducted.

### Analysis of the interactome

**Figure 6:**
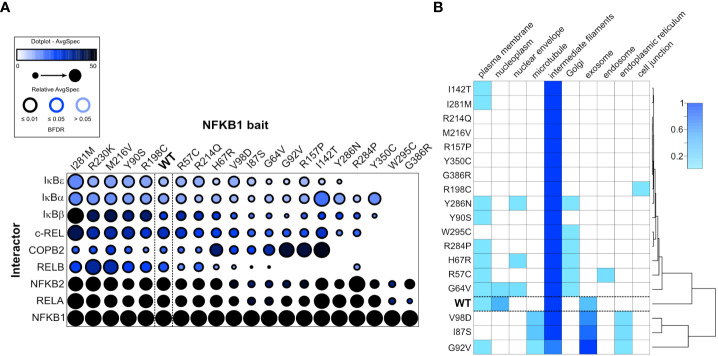
NFKB1 missense variants cause protein interaction defects and subcellular mis-localization. **(A)** Dotplot of selected p105/p50 interactors illustrates the protein interaction changes induced by single amino acid variants. The order of the NFKB1 baits is based on hierarchical clustering. **(B)** The heatmap shows the molecular context of NFKB1 variants based on the annotation score of the MS-microscopy system. The cluster tree (right side) indicates common activities.

To circumvent the intrinsic drawbacks associated with protein overproduction upon transient transfection, we employed a stably transfected cell line model with tetracycline-inducible expression of wildtype or mutant p105 (6). We then tested, whether *NFKB1* missense variants cause alterations of protein-protein interactions. Therefore, we analyzed the interactome of wildtype NFKB1 and the NFKB1 missense variants using the used MAC-tag approach (31, 32), which enables the analysis of both stable (AP-MS) and transient proximal interactions (BioID). The NF-κB family members (NFKB2, RELA, RELB and c-REL) and the inhibitors-of-NF-κB (IκB`s, including IκBα, IκBβ, IκBε) form the majority of the p105/p50 stable interactome detected by the AP-MS. Compared to the wildtype NFKB1, several missense variants showed clear and quantitative interaction chances (**Figure 6A**), particularly weakened or lost interaction with RELB. Mutations in the N-terminal part of the Rel Homology domain (G64V, H67R, I87S, G92V) displayed moderately reduced interactions with RELB. Missense variants residing in the central or the C-terminal part of the Rel-homology domain (I142T, R157P, R284P, Y286N, W295C and G386R) showed mostly a complete loss-of-interaction with RELB and IκBε. The two most proximally localized missense variants (W295C and G386R) additionally lost their ability to interact with IκBα, IκBβ and c-REL. Many of the missense variants which caused weakened or complete loss-of-interaction with RELB, additionally showed reduced interactions with NFKB2 and RELA.

The BioID interactomes were used for MS-microscopy based analysis of the NFKB1 localization (**Figure 6B**). In nearly all cases, the highest localization score was given for intermediate filaments and some also had high scoring for exosome. The wildtype and G64V showed localization also in the nucleoplasm. Plasma membrane, ER and Golgi were also marked as possible localizations for many of the variants. Interestingly, the mutations residing on the C-terminal part of the Rel-homology domain clearly clustered as their own entity, possibly suggesting distinct cellular localization compared to the wildtype NFKB1.

### Clinical summary

Detailed clinical information is provided in the Supplement of this article but was not available for all individuals carrying the analyzed *NFKB1* variants (**Supplementary Table 3** and case reports). As previously described (10), the phenotype and the age at onset (median: 7.5 years; age range: 0-61 years) of the symptoms in patients carrying pathogenic *NFKB1* variants are highly variable. In line with this observation, the age at PID diagnosis in the current study ranged from 1 to 69 years of age. However, the median age at onset was 23 years, possibly indicating a delay in the PID diagnosis. No significant differences in the disease phenotype or the age at onset of the symptoms were identified among the individuals carrying *NFKB1* variants with a defined functional defect and those without a specified functional defect.

Clinical information was available for 15 (13 symptomatic and 2 asymptomatic) of the 16 individuals carrying deleterious *NFKB1* missense variants (I87S, G92V, V98D, I142T, R157P, R284P, Y286N, W295C, Y350C). Seven of the 13 affected individuals presented with symptoms early in life (median 14 years) including lymphoproliferation and autoimmunity. Eleven of them were diagnosed with CVID, which explains the high incidence of bacterial infections in this group. Splenomegaly (n=7) and autoimmune phenomena (n=7) were also described. All of the CVID patients are under IgG replacement. One of the two unaffected carriers is a teenager, who may develop disease symptoms at an older age. The two remaining patients with a deleterious *NFKB1* missense variant had splenomegaly and autoimmune cytopenias as common features and started at a young age with the first symptoms (1.5 and 12 years, respectively). Interestingly, the patient carrying the G92V variant had hypogammaglobulinemia and required IgG replacement while the patient carrying the I142T had slightly elevated levels of IgG and normal levels of the other immunoglobulins. He received IgG replacement with minimal response in his pancytopenia.

Two of the four individuals carrying variants with loss of the DNA-binding function (R57C, G64V) are still unaffected at the age of 18 and 75 respectively. The other two started with recurrent upper respiratory infections in childhood and were later diagnosed with CVID, requiring IgG replacement.

Nine of the eleven patients carrying the previously described pathogenic H67R variant (which however had ambiguous results in our tests) belong to the same family (6). Four of these were diagnosed with CVID and required IgG replacement therapy. Consistent with hypogammaglobulinemia, these patients had recurrent respiratory infections. In this family, skin infections and sepsis were observed and immune dysregulation, autoimmunity and gastrointestinal involvement were common features. Aphthous ulcers were present in six of these individuals, as well as in both members (father and daughter) of the second family carrying this variant. Both were diagnosed with antibody deficiency and had recurrent respiratory infections, with good response to IgG substitution. The father was additionally diagnosed with chronic lymphocytic leukemia.

Four out of the ten individuals carrying variants with ambiguous functional test results (H67Y, Y90S, R214Q, M216V, A245V, I281M) were diagnosed with CVID. Three did not have a clinically evident immunodeficiency and were considered to be unaffected. Hypogammaglobulinemia was detected in eight subjects. The clinical manifestations were highly variable including infections of the upper respiratory tract, the gastrointestinal tract and the skin. Autoimmunity was also described in this group. Two individuals had autoimmune cytopenias, one had autoimmune thyroiditis and one presented with psoriasis.

The *NFKB1* variants, which showed only a minor functional defect or for which no defect could be specified, were identified in patients with CVID (n=11), antibody deficiency (n=6), autoinflammatory disorder (n=2), CID (n=1) or in unaffected individuals (n=4). Two of the variants, for which no defect could be specified were somatic variants, which have previously been observed in samples of speckled leukemia and cervical cancer, respectively. The 20 symptomatic individuals in this subgroup presented with respiratory tract infections (n=16), skin infections (n=5), gastrointestinal infections (n=4), sepsis (n=2), meningitis (n=2), autoimmune cytopenia (n=6) and autoimmune thyroiditis (n=2). Twelve of the neutral variants were initially observed in control cohorts of the NIHR BioResource-Rare Diseases study (7). As we do not have clinical information on these subjects, they were not counted as unaffected individuals.

## Discussion

Pathogenic *NFKB1* variants are associated with highly variable disease phenotypes, among which antibody deficiency, hyperinflammatory lesions, and autoimmune phenomena are the most frequent manifestations, with a very variable age of onset of symptoms (10). Considering that most of the known pathogenic variants – mainly comprising severe protein truncations - cause p105/p50 haploinsufficiency, a genotype-phenotype correlation was expected but could not be demonstrated. On the contrary, not only the expressivity of mutations within families but also their penetrance is highly variable, pointing to additional factors influencing disease activity.

### Accelerated decay of mutant p105 precursors prevents generation of dysfunctional NF-κB transcription factors

Here, we show that mutant p105 precursor proteins, harboring a deleterious missense variant within their Rel-homology domain, undergo immediate decay (without first being processed to p50). This appears to be an eligible mechanism to protect the cells from generating dysfunctional NF-κB transcription factors (19, 23). While the C-terminal half of the p105 precursor comprises the inhibitory IκB-like domains, the N-terminal p50 half harbors the multi-functional Rel-homology domain. The latter functions are only employed by the mature p50, including the nuclear localization sequence and the domains for DNA-binding, dimerization with other NF-κB proteins and IκB binding.

### Cohort studies and mutation testing draw the silhouette of NFKB1 disease

Our standard *in vitro* transfection model predominantly assesses the activities of mature p50 while the functions of p105 largely remain untested. We therefore restricted our analyses to the N-terminal amino acid variants, which all were delineated from the coding DNA sequence changes identified by genetic testing. *In silico* analyses predicted 19 damaging variants, of which 9 were experimentally confirmed to have a decaying defect while two variants had a loss-of-DNA-binding defect (**Table 1**; **Supplementary Table 1**). Two of the variants gained inconclusive results, four had unclear results with weak evidence and only two were tested neutral. Among the 28 variants which had benign or weak *in silico* predictions, 26 had no effects in our test and the remaining two had only minor differences compared to the controls. Therefore, pathogenicity predictions correlated well with our experimental data, particularly when the prediction was “benign”.

Two of the three missense variants classified as pathogenic in the Tuijnenburg study (7) were confirmed to have a decaying defect (I87S, V98D) whereas the impact of the I281M variant remained unclear in our tests. In their study, the authors generally considered variants to have a near-zero probability of pathogenicity when they occurred in both their CVID cohort and in (rare diseases) control cohorts. In full agreement with this assessment, which included eleven missense variants localizing within the N-terminal p50 half (**Table 1**), none of these variants showed a deleterious effect in our functional analyses. However, whether these variants are indeed unable to cause NF-κB signaling defects has yet to be confirmed experimentally (19).

The Lorenzini study lists 27 missense variants affecting the N-terminal p50 half, including five variants (H67R, I87S, V98D, R157P, I281M) with a known protein defect, one (G386R) with an unknown protein defect (6–8), and 21 “candidate variants” which have previously not been reported (10; **Table 1**). Lorenzini et al. tested five of the newly described variants using a classical luciferase reporter assay, which enabled the identification of one missense variant causing a protein defect (R57C). In the current study, we tested or re-analyzed all 27 variants of the Lorenzini report and found nine variants (including four previously reported mutants) to have a deleterious effect, seven had variable results, but eleven variants were indistinguishable from controls (**Table 1**; **Supplementary Table 1**). Therefore, our observations support the concept of the Lorenzini study to consider *NFKB1* variants discovered in patients with immune dysregulation, particularly the rare heterozygous variants with a population frequency of <0.01%, as “possible mutations” unless proven otherwise.

In a recent report, Li et al. analyzed 170 N-terminal missense variants for p50-dependent reporter gene activation following co-transfection of constructs encoding the mutant precursor proteins together with a homodimerization-defective RelA into p105/p50-deficient HEK293T cells (18). The study also included 36 of the 47 variants analyzed in our report (**Table 1**; **Supplementary Table 1**) and, consistent with our results, identified the protein-decaying variants as loss-of-function (I87S; V98D; I142T; R157P; W295C; Y350C) or hypomorphic (Y286N) variants. The two non-decaying, DNA-binding-deficient variants which had opposite effects in our reporter assays, were assessed in the Li et al. study as loss-of-function (R57C) and hypomorphic (G64V), respectively, and thus, in both studies, indicate the presence of a unique defect. Similarly, the two non-decaying variants H67Y and H67R, which gained normal or reduced reporter activities, respectively, were both hypomorphic in the Li study. Several variants were inconspicuous in the Li et al. study but had variable effects in our assays (Y90S; N103D; R214Q; M216V; A245V; I281M) which could not be further specified. Thus, both approaches were equally efficient in detecting the deleterious (decaying, loss-of-function and hypomorphic) p50 missense variants, yet, have a limited capacity to characterize less apparent defects. This might particularly apply to missense variants localizing to the C-terminal half of p105 (data not shown), which only affect the precursor proteins and therefore might be indistinguishable from overexpressed wildtype p105 in p50-dependent assays (18).

### The search for undisclosed protein defects

Due to the limited sensitivity of our assays, we were unable to confirm or exclude hypomorphic functional defects for several of the missense variants (Y90S, N103D, R214Q, M216V, A245V, I281M, G386R). Here we obtained ambiguous results (data not shown), which might partially depend on even minor changes in experimental conditions. For instance, the amount of the co-transfected RelA was found to be critical (**Supplementary Figure 2**) since it can mask the p50-mediated inhibitory effect in our reporter assays (23). A further drawback is the “snap-shot character” determined by the time-point(s) of the analyses, which disregards the dynamic parameters such as p105 precursor-processing or p50 nuclear shuttling. In addition, overexpression of p105 and particularly the experimental maneuver to directly overload the nuclei with mutant p50 (thereby by-passing the requirement of producing p50 from its precursor), yet a good attempt to “insulate” the mutant proteins from the context of the NF-κB signaling network, is a highly artificial approach. Therefore, stimulus-inducible or on-off-switchable B and T cell models would be a huge advantage and would resemble the physiological conditions and the cell type specific effects more closely than our transient HEK293T transfection system.

We do not consider rare variants, which were indistinguishable from controls in our assays as necessarily non-pathogenic. For instance, although we analyze mutant proteins, the primary defect of exonic sequence variants might originate from splicing defects, rather than from amino acid changes. In fact, a recent report described a silent *NFKB1* variant (c.705G>A) leading to activation of a cryptic splice site (33). Three of the single base-pair changes analyzed in our study are located within the first or last codon of the respective exon (c.115A>G, T39A; c.260T>G, I87S; c.406G>A, G136S) and could therefore possibly interfere with the consensus splice sites. In addition, several variants could potentially lead to splicing defects e.g. due to cryptic splice site activation (delineated from the AG-GT rule), which however has not been pursued further.

Western blot data with patient-derived cells were also available from previous studies (**Supplementary Table 3**) for four of the decaying variants (I87S; V98D; R157P; Y350C), which consistently demonstrated reduced p105/p50 expression levels, confirming (haplo)insufficient conditions (7, 8, 23). Whereas the role of the I281M remained unresolved, cells heterozygous for the non-decaying variant H67R had normal p105/p50 levels (6).

A future task will be to refine the available experimental models to enable the characterization of non-deleterious effects such as altered signaling dynamics, impaired binding partner interactions or defects of post-translational modification sites. For instance, the G430E variant, which was neutral in all of our tests, but is known as a somatic mutation in cervical cancer (25), might affect the Lys432 acetylation site. Other examples include the post-translational modification sites Cys62, Ser66, Ser329 and Ser338 (34, 35) which might be affected by amino acid changes in close proximity. Moreover, the neutral variant R192W showed a slightly reduced size of the EMSA band, yet because of unknown reasons. However, we cannot exclude posttranslational modifications to cause such effects. On the other hand, the H67Y variant, which has been reported to cause delayed p50 nuclear entry (6), showed less DNA-binding upon transfection of p105 (but not of p50) and reduced reporter activation upon transfection of p105 or p50, although expression and localization of both proteins were normal.

Therefore, we assume at least some of the minor abnormalities detected in our assays to represent a potentially pathogenic effect. A careful characterization of these variants, applying functional tests with higher sensitivity, will be a prerequisite to complete the emerging picture of the NFKB1 pathophysiology.

### Genetic counseling of *NFKB1* variant carriers - impact of this study for clinicians

Individuals with variants in *NFKB1* may fall into one of three distinct categories. (1) Pathogenic variants proved to be deleterious for cell biology, such as the nine decaying and the two DNA-binding deficient variants we present in this study. These patients should be counseled regarding disease penetrance and expressivity, family planning with an autosomal dominant trait, and first-degree relatives should be offered genetic screening and counceling. (2) If the variant in *NFKB1* has a frequency in the respective population of >0.0001 (>0.01%; >1 in 10.000) we do not advise further work-up. (3) If the *NFKB1* variant has a population frequency of <0.0001 (<0.01%; <1 in 10.000), but functional testing was either not done or inconclusive or revealed results similar to healthy controls, we advise to perform family screening of the variant in first degree relatives and a referral to a tertiary center for IEI for a further work-up and longitudinal follow-up. *NFKB1* variants suggesting a splice defect need to be verified by cDNA sequencing.

## Data availability statement

The original contributions presented in the study are included in the article/**Supplementary Material**. Further inquiries can be directed to the corresponding author.

## Ethics statement

This study was carried out in accordance with the recommendations for studies with human subjects of the scientific committee at the University Medical Center of Freiburg. All physicians confirmed that their patients had signed an informed consent under local ethics-approved protocols and in accordance with the Declaration of Helsinki. The study protocol was approved by the ethics committee of the University Medical Center of Freiburg (Approval No. 295/13_200149 and 93/18_191111). No financial incentive was provided, neither to the patients nor the contributing physicians. Data was reported pseudo-anonymized, and physician-to-physician contact allowed to communicate treatment results and advice. Written informed consent was obtained from the individuals and the minor`s legal guardian for the publication of any potentially identifiable data included in this article.

## Author contributions

MF contributed the experiments shown in **Figures 1**−**5** and the Supplement, provided the tables and wrote the experimental part of the manuscript. MK and MV contributed the experiments shown in **Figure 6**. SP-C, NC-O, and BG wrote the clinical sections of the manuscript. SP-C, NC-O, HA, FA, LA, DB, SB, JC, GD, AF, LH, LGH, TK, RK, SOS, KS, CS, SS, NV, JW, EW-C, SG, KW, and BG cared for patients and/or collected, analyzed and summarized clinical, diagnostic and immunological information, and/or provided case reports. BG coordinated and supervised the study and revised the manuscript. All authors reviewed and approved the manuscript.

## Funding

This study was supported by the Deutsche Forschungsgemeinschaft (DFG, German Research Foundation) under Germany’s Excellence Strategy (CIBSS – EXC-2189 - Project ID 390939984 and RESIST - EXC-2155 - Project ID 39087428) and by the collaborative research center SFB1160/IMPATH to BG. BG received financial support from the E-rare programme of the EU, managed by the Deutsche Forschungsgemeinschaft (DFG), grant code GR1617/14-1/iPAD and from the ”Netzwerke Seltener Erkrankungen” by the German Federal Ministry of Education and Research (Bundesministerium für Bildung und Forschung, BMBF) through a grant to the German Auto-Immunity Network (GAIN), grant code 01GM1910A, and a collaborative research grant between Merck and UKL-FR (grant# ZVK2018073002). The article processing charge was funded by the Baden-Wuerttemberg Ministry of Science, Research and Art and the University of Freiburg in the funding programme Open Access Publishing. AFF was supported by the Division of Intramural Research (DIR) of the National Institute of Allergy & Infectious Diseases, National Institutes of Health. FA and KW received support by the German Federal Ministry of Education and Research (BMBF) through a grant to the German Auto-Immunity Network (GAIN), grant codes 01GM1910E and 01GM1910A, respectively.

## Acknowledgments

The authors are deeply grateful to all affected individuals, their family members and all healthy volunteers who participated in this study. We thank Pavla Mrovecova, Katrin Hübscher, Mary Buchta, Hanna Haberstroh and Anastasija Maks for excellent technical assistance. We highly appreciate the continuous support from the CCI Advanced Diagnostics Unit and the CCI-Biobank, a partner biobank of the University Medical Center Freiburg and Medical Faculty “Center for Biobanking – FREEZE”. We wish to acknowledge the Lighthouse Core Facility for their assistance with microscopy.

## Conflict of interest

The authors declare that the research was conducted in the absence of any commercial or financial relationships that could be construed as a potential conflict of interest.

## Publisher’s note

All claims expressed in this article are solely those of the authors and do not necessarily represent those of their affiliated organizations, or those of the publisher, the editors and the reviewers. Any product that may be evaluated in this article, or claim that may be made by its manufacturer, is not guaranteed or endorsed by the publisher.

## Abbreviations

CVID, Common variable Immunodeficiency; EGFP, Enhanced green fluorescent protein; EMSA, Electrophoretic mobility shift assay; NFKB1, Nuclear factor kappa B subunit 1; NF-κB, Nuclear factor ‘kappa-light-chain-enhancer’ of activated B-cells; NLS, Nuclear localization sequence; PID, Primary immunodeficiency; RHD, Rel-homology domain; VUS, Variant of uncertain significance; WES, Whole Exome Sequencing.
