# Supplementary material for: Detrimental NFKB1 missense variants affecting the Rel-homology domain of p105/p50
Source: Front Immunol. 2022 Aug 29;13:965326. doi: 10.3389/fimmu.2022.965326 (PMC9465457; doi:10.3389/fimmu.2022.965326)
Supplement: Supplementary file 1 [file DataSheet_1.pdf]

Supplementary Table 1: Variant effect predictions

Position on Chr. 4 (GRCh38 coordinates)	exon	cDNA change	Protein change (HGVS)	Protein change (single letter code)	Grantham score (Mutation Taster)	Mutation Taster tree vote	Mutation Taster category	SIFT scores ENSEMBL Variant Effect Predictor & CADD	SIFT category ENSEMBL Variant Effect Predictor & CADD	ENSEMBL Variant Effect Predictor scores	ENSEMBL Variant Effect Predictor category	PolyPhen-2 / PolyPhen-2 (batch) values (HumVar)	PolyPhen-2 / PolyPhen-2 (batch) category (HumVar)	CADD score	REVEL scores ENSEMBL Variant Effect Predictor	REVEL category ENSEMBL Variant Effect Predictor	gnomAD allele count	pathogenic effect predictions (synopsis)	functional defect (synopsis; see main text)	assessment in Li et al.2021	Reference
102525534	2	c.16C>T	p.Pro6Ser	P6S	74	5 / 95	benign	0.63	tolerated	12.29	benign	0 / 0.001	benign	12.29	0.014	likely benign	0	no	no	—	Tuijnenburg et al., 2018
102529902	3	c.106G>A	p.Ala36Thr	A36T	58	9 / 91	benign	0.38	tolerated	12.71	benign	0 / 0.001	benign	12.71	0.050	likely benign	0	no	no	neutral	Lorenzini et al., 2020
102529911	3	c.115A>G	p.Thr39Ala	T39A	58	11 / 89	benign	0.04	deleterious	20.6	benign	0.031	benign	20.60	0.047	likely benign	11 (het)	no	no	neutral	Tuijnenburg et al., 2018; Li et al., 2021
102533857	4	c.131A>G	p.Tyr44Cys	Y44C	194	39 / 61	benign	0.03	deleterious	23.3	benign	0.05 / 0.114	benign	23.30	0.133	likely benign	0	no	no	—	Tuijnenburg et al., 2018
102537867	5	c.169C>T	p.Arg57Cys	R57C	180	52 / 48	deleterious	0	deleterious	32	probably damaging	0.997 / 1	probably damaging	32	0.793	likely disease causing	0	**yes**	**yes**	LOF	Lorenzini et al., 2020; Rojas-Restrepo et al., 2021
102537889	5	c.191G>T	p.Gly64Val	G64V	109	76 / 24	deleterious	0	deleterious	27.9	probably damaging	0.999	probably damaging	27.90	0.547	likely disease causing	0	**yes**	**yes**	hypomorphic	Lorenzini et al., 2020
102537897	5	c.199C>T	p.His67Tyr	H67Y	83	42 / 58	benign	0	deleterious	23.3	benign	0.418 / 0.482	benign / possibly damaging	23.30	0.339	likely benign	0	weak	**yes**/unclear	hypomorphic	Lorenzini et al., 2020
102537898	5	c.200A>G	p.His67Arg	H67R	29	83 / 17	deleterious	0	deleterious	25.2	probably damaging	0.916 / 0.934	probably damaging	25.20	0.527	likely disease causing	0	**yes**	**yes**	hypomorphic	Kaustio et al., 2017; Lorenzini et al., 2020
102566988	6	c.260T>G	p.Ile87Ser	I87S	142	97 / 3	deleterious	0	deleterious	32	probably damaging	0.988 / 0.977	probably damaging	32.00	0.666	likely disease causing	0	**yes**	**yes**	LOF	Tuijnenburg et al., 2018; Lorenzini et al., 2020
102566997	6	c.269A>C	p.Tyr90Ser	Y90S	144	71 / 29	deleterious	0	deleterious	27.2	possibly damaging	0.787 / 0.984	possibly / probably damaging	27.20	0.742	likely disease causing	0	**yes**	**yes**/unclear	neutral	Lorenzini et al., 2020; Rojas-Restrepo et al., 2021
102567003	6	c.275G>T	p.Gly92Val	G92V	109	88 / 12	deleterious	0.02	deleterious	28.0	probably damaging	0.997 / 0.982	probably damaging	28.00	0.796	likely disease causing	0	**yes**	**yes**	—	this study
102567021	6	c.293T>A	p.Val98Asp	V98D	152	99 / 1	deleterious	0	deleterious	27.6	probably damaging	0.983 / 0.999	probably damaging	27.60	0.754	likely disease causing	0 (altern 1)	**yes**	**yes**	LOF	Tuijnenburg et al., 2018; Lorenzini et al., 2020
102567035	6	c.307A>G	p.Asn103Asp	N103D	23	28 / 72	benign	0.02	deleterious	23.3	benign	0.011 / 0.054	benign	23.30	0.069	likely benign	0	no	no/unclear	—	this study
102567134	6	c.406G>A	p.Gly136Ser	G136S	56	14 / 86	benign	0.93	tolerated	17.17	benign	0.001 / 0.003	benign	17.17	0.039	likely benign	19 (het)	no	no	neutral	this study; Li et al., 2021
102576887	7	c.419T>A	p.Leu140Gln	L140Q	113	69 / 31	deleterious	0	deleterious	28.0	probably damaging	1	probably damaging	28.00	0.667	likely disease causing	0	**yes**	no	—	Govindarajan et al., 2016
102576893	7	c.425T>C	p.Ile142Thr	I142T	89	95 / 5	deleterious	0	deleterious	27.5	probably damaging	1	probably damaging	27.50	0.779	likely disease causing	0	**yes**	**yes**	LOF	Duan & Feanny 2019
102576938	7	c.470G>C	p.Arg157Pro	R157P	103	99 / 1	deleterious	0	deleterious	29.3	probably damaging	0.998 / 1	probably damaging	29.30	0.674	likely disease causing	0	**yes**	**yes**	LOF	Schröder et al., 2019; Lorenzini et al., 2020
102577024	7	c.556G>T	p.Asp186Tyr	D186Y	160	33 / 67	benign	0.04	deleterious	26.5	possibly damaging	0.866 / 0.697	possibly damaging	26.50	0.141	likely benign	1 (het)	weak	no	neutral	Lorenzini et al., 2020
102578883	8	c.574C>T	p.Arg192Trp	R192W	101	30 / 70	benign	0.01	deleterious	26.6	probably damaging	0.972 / 0.925	probably damaging	26.60	0.219	likely benign	0 (altern 1)	weak	no/unclear	—	Tuijnenburg et al., 2018
102578895	8	c.586C>G	p.Leu196Val	L196V	32	14 / 86	benign	0.5	tolerated	14.65	benign	0 / 0.001	benign	14.65	0.020	likely benign	0	no	no	—	this study
102578901	8	c.592C>T	p.Arg198Cys	R198C	180	48 / 52	benign	0.06	tolerated	24.7	possibly damaging	0.742 / 0.983	possibly /probably damaging	24.70	0.223	likely benign	2 (het)	weak	no/unclear	neutral	Lorenzini et al., 2020
102578902	8	c.593G>A	p.Arg198His	R198H	29	31 / 69	benign	0.21	tolerated	21.9	benign	0.005 / 0.013	benign	21.90	0.047	likely benign	109 (het)	no	no	neutral	Tuijnenburg et al., 2018; Li et al., 2021
102578913	8	c.604C>A	p.Leu202Met	L202M	15	12 / 88	benign	0.25	tolerated	15.53	benign	0.011 / 0.27	benign	15.53	0.049	likely benign	1 (het)	no	no	—	Tuijnenburg et al., 2018
102578950	8	c.641G>A	p.Arg214Gln	R214Q	43	65 / 35	deleterious	0	deleterious	31	probably damaging	0.998 / 0.999	probably damaging	31.00	0.649	likely disease causing	0 (altern 2)	**yes**	**yes**/unclear	neutral	Lorenzini et al., 2020; Rojas-Restrepo et al., 2021
102578955	8	c.646A>G	p.Met216Val	M216V	21	58 / 42	deleterious	0.04	deleterious	24.2	possibly damaging	0.839 / 0.982	possibly / probably damaging	24.20	0.317	likely benign	0 (altern 2)	**yes**	**yes**/unclear	neutral	Lorenzini et al., 2020; Rojas-Restrepo et al., 2021
102578998	8	c.689G>A	p.Arg230Lys	R230K	26	42 / 58	benign	0.04	deleterious	24.0	benign	0.111 / 0.676	benign / possibly damaging	24.00	0.177	likely benign	0	weak	no	neutral	Lorenzini et al., 2020
102579000	8	c.691C>T	p.Arg231Cys	R231C	180	15 / 85	benign	0.07	tolerated	24.0	possibly damaging	0.796 / 0.972	possibly / probably damaging	24.00	0.208	likely benign	14 (het) (altern 3; 11)	weak	no	neutral	Tuijnenburg et al., 2018; Anim et al., 2021
102580538	9	c.734C>T	p.Ala245Val	A245V	64	77 / 23	deleterious	0	deleterious	25.8	probably damaging	0.968 / 0.91	probably damaging	25.80	0.420	likely benign	no (altern 1)	**yes**	**yes**/unclear	neutral	Lorenzini et al., 2020
102580540	9	c.736C>A	p.Pro246Thr	P246T	38	85 / 15	deleterious	0	deleterious	24.0	possibly damaging	0.804 / 0.708	possibly damaging	24.00	0.334	likely benign	0	**yes**	no	neutral	Lorenzini et al., 2020
102582873	10	c.843C>G	p.Ile281Met	I281M	10	49 / 51	deleterious	0	deleterious	23.5	probably damaging	1	probably damaging	23.50	0.749	likely disease causing	0	**yes**	**yes**/unclear	neutral	Tuijnenburg et al., 2018; Lorenzini et al., 2020
102582881	10	c.851G>C	p.Arg284Pro	R284P	103	94 / 6	deleterious	0	deleterious	32	probably damaging	0.993 / 1	probably damaging	32.00	0.953	likely disease causing	0	**yes**	**yes**	—	this study
102582886	10	c.856T>A	p.Tyr286Asn	Y286N	142	89 / 11	deleterious	0	deleterious	28.1	probably damaging	0.997 / 1	probably damaging	28.10	0.942	likely disease causing	0	**yes**	**yes**	hypomorphic	Lorenzini et al., 2020
102582915	10	c.885G>C	p.Trp295Cys	W295C	215	87 / 13	deleterious	0	deleterious	31	probably damaging	1	probably damaging	31.00	0.693	likely disease causing	0	**yes**	**yes**	LOF	Lorenzini et al., 2020
102584719	11	c.965T>C	p.Ile322Thr	I322T	89	26 / 74	benign	0.24	tolerated	20.6	benign	0.001 / 0.025	benign	20.60	0.100	likely benign	3 (het) (altern 2)	no	no	neutral	Tuijnenburg et al., 2018
102584721	11	c.967A>T	p.Asn323Tyr	N323Y	143	94 / 6	deleterious	0.01	deleterious	23.3	benign	0.26 / 0.182	benign	23.30	0.272	likely benign	no	weak	no	—	Christiansen et al., 2020
102584732	11	c.978A>C	p.Lys326Asn	K326N	94	99 / 1	deleterious	0	deleterious	22.2	possibly damaging	0.874 / 0.945	possibly / probably damaging	22.20	0.169	likely benign	1459 (het)	weak	no	neutral	Lorenzini et al., 2020; Li et al., 2021
102584758	11	c.1004G>A	p.Arg335Gln	R335Q	43	14 / 86	benign	0.01	deleterious	24.2	benign	0.307 / 0.503	benign / possibly damaging	24.20	0.145	likely benign	49 (het)	weak	no	neutral	Lorenzini et al., 2020
102584803	11	c.1049A>G	p.Tyr350Cys	Y350C	194	85 / 15	deleterious	0	deleterious	26.7	probably damaging	1	probably damaging	26.70	0.617	likely disease causing	0	**yes**	**yes**	LOF	Lorenzini et al., 2020; Fliegauf et al., 2021
102593473	12	c.1115C>T	p.Ser372Leu	S372L	145	11 / 89	benign	0.22	tolerated	22.8	benign	0.218	benign	22.80	0.091	likely benign	1 (het)	no	no	neutral	Lorenzini et al., 2020
102593484	12	c.1126G>A	p.Gly376Ser	G376S	56	20 / 80	benign	0.05	tolerated	23.8	possibly damaging	0.738	possibly damaging	23.80	0.147	likely benign	12 (het)	no	no	neutral	Lorenzini et al., 2020
102593487	12	c.1129G>A	p.Gly377Ser	G377S	56	9 / 91	benign	0.01	deleterious	23.5	benign	0.116	benign	23.50	0.296	likely benign	5 (het)	no	no	neutral	Tuijnenburg et al., 2018; Li et al., 2021
102593505	12	c.1147G>T	p.Ala383Ser	A383S	99	15 / 85	benign	0.07	tolerated	14.37	benign	0.107	benign	14.37	0.023	likely benign	0	no	no	neutral	Lorenzini et al., 2020
102593514	12	c.1156G>A	p.Gly386Arg	G386R	125	10 / 90	benign	0.23	tolerated	24.3	probably damaging	0.909	probably damaging	24.30	0.200	likely benign	5 (het)	weak	no/unclear	neutral	Tuijnenburg et al., 2018; Lorenzini et al., 2020; Li et al., 2021
102593535	12	c.1177G>A	p.Gly393Ser	G393S	56	16 / 84	benign	0.29	tolerated	14.97	benign	0.322	benign	14.97	0.217	likely benign	3 (het)	no	no	neutral	Lorenzini et al., 2020
102594895	13	c.1214A>G	p.Tyr405Cys	Y405C	194	14 / 86	benign	0.04	deleterious	24.7	possibly damaging	0.843	possibly damaging	24.70	0.145	likely benign	3 (het)	no	no	neutral	this study
102594907	13	c.1226A>T	p.His409Leu	H409L	99	4 / 96	benign	0.6	tolerated	15.57	benign	0.048	benign	15.57	0.059	likely benign	1 (het)	no	no	neutral	Tuijnenburg et al., 2018
102594970	13	c.1289G>A	p.Gly430Glu	G430E	98	16 / 84	benign	0.02	deleterious	26.2	probably damaging	0.991	probably damaging	26.20	0.172	likely benign	0	weak	no	—	Yang et al., 2017

Supplementary Table 2: Expression levels of p105 and p50 variants and reporter activity values

Protein change (single letter code)	p105 level (Reporter assay)	Reporter activity p105	p50 level (Reporter assay)	Reporter activity p50
P6S	nd	nd	10.13 ± 2.79 (↔)	3.49 ± 0.89 (↔)
A36T	nd	nd	10.37 ± 1.93 (↔)	3.17 ± 0.25 (↔)
T39A	nd	nd	9.79 ± 2.41 (↔)	2.90 ± 0.85 (↔)
Y44C	nd	nd	10.35 ± 2.30 (↔)	3.58 ± 0.73 (↔)
R57C	4.45 ± 0.20 (↔)	6.67 ± 0.05 **(↓)**	7.24 ± 0.99 **(↓)**	1.20 ± 0.01 **(↓)**
G64V	nd	nd	12.43 ± 1.22 (↔)	4.69 ± 1.27 **(↑)**
H67Y	nd	nd	10.26 ± 1.03 (↔)	1.79 ± 0.31 **(↓)**
H67R	4.19 ± 0.62 (↔)	2.74 ± 0.40 **(↓)**	11.42 ± 0.56 (↔)	3.46 ± 0.27 (↔)
I87S	2.23 ± 0.18 **(↓)**	2.95 ± 0.65 **(↓)**	3.47 ± 0.76 **(↓)**	5.89 ± 0.77 **(↑)**
Y90S	3.21 ± 0.04 **(↓ ?)**	5.71 ± 1.71 (↔)	6.06 ± 1.46 **(↓)**	3.27 ± 1.53 (↔)
G92V	nd	nd	2.91 ± 0.43 **(↓)**	4.89 ± 0.77 **(↑)**
V98D	2.30 ± 0.20 **(↓)**	0.95 ± 0.10 **(↓)**	3.12 ± 0.23 **(↓)**	2.26 ± 0.19 **(↓?)**
N103D	4.15 ± 0.19 (↔)	5.36 ± 0.74 (↔)	11.30 ± 1.00 (↔)	4.45 ± 1.61 **(↑)**
G136S	4.33 ± 0.39 (↔)	4.89 ± 0.57 (↔)	11.90 ± 0.95 (↔)	6.32 ± 1.45 **(↑)**
L140Q	nd	nd	10.05 ± 0.68 (↔)	3.86 ± 0.34 **(↑?)**
I142T	nd		4.18 ± 0.07 **(↓)**	9.01 ± 0.73 **(↑)**
R157P	2.49 ± 0.24 **(↓)**	1.25 ± 0.14 **(↓)**	4.41 ± 1.03 **(↓)**	4.42 ± 1.42 **(↑)**
D186Y	nd	nd	11.73 ± 2.39 (↔)	3.45 ± 1.15 (↔)
R192W	nd	nd	12.21 ± 2.40 (↔)	3.31 ± 0.72 (↔)
L196V	nd	nd	10.72 ± 2.73 (↔)	3.30 ± 1.27 (↔)
R198C	4.00 ± 0.26 (↔)	6.03 ± 0.65 (↔)	10.94 ± 2.26 (↔)	3.91 ± 0.93 **(↑ ?)**
R198H	nd	nd	10.74 ± 1.74 (↔)	2.67 ± 0.70 (↔)
L202M	nd	nd	11.16 ± 2.54 (↔)	2.87 ± 0.91 (↔)
R214Q	4.00 ± 0.32 (↔)	4.80 ± 0.78 **(↓ ?)**	8.38 ± 1.17 **(↓)**	2.94 ± 0.96 (↔)
M216V	3.90 ± 0.17 (↔)	4.46 ± 0.46 **(↓ ?)**	8.15 ± 1.38 **(↓)**	1.21 ± 0.42 **(↓)**
R230K	3.92 ± 0.31 (↔)	5.55 ± 0.98 (↔)	11.67 ± 2.96 (↔)	3.16 ± 1.36 (↔)
R231C	nd	nd	11.82 ± 2.52 (↔)	3.71 ± 1.19 (↔)
A245V	3.68 ± 0.26 (↔)	6.29 ± 0.36 (↔)	11.24 ± 2.53 (↔)	5.26 ± 2.17 **(↑)**
P246T	3.83 ± 0.23 (↔)	5.57 ± 0.31 (↔)	11.82 ± 3.22 (↔)	3.55 ± 1.34 (↔)
I281M	3.66 ± 0.13 (↔)	4.73 ± 0.46 **(↓ ?)**	8.83 ± 2.13 **(↓?)**	4.57 ± 1.88 **(↑)**
R284P	nd	nd	2.93 ± 0.66 **(↓)**	9.78 ± 1.56 **(↑)**
Y286N	1.61 ± 0.10 **(↓)**	2.56 ± 0.37 **(↓)**	4.57 ± 0.85 **(↓)**	9.77 ± 2.56 **(↑)**
W295C	1.22 ± 0.11 **(↓)**	2.17 ± 0.41 **(↓)**	3.05 ± 0.33 **(↓)**	8.86 ± 2.15 **(↑)**
I322T	nd	nd	11.98 ± 0.77 (↔)	2.93 ± 0.60 (↔)
N323Y	nd	nd	12.08 ± 0.75 (↔)	3.84 ± 1.60 (↔)
K326N	nd	nd	11.38 ± 0.70 (↔)	3.86 ± 1.27 (↔)
R335Q	nd	nd	12.24 ± 0.52 (↔)	3.62 ± 0.44 (↔)
Y350C	1.30 ± 0.07 **(↓)**	3.15 ± 0.85 **(↓)**	3.58 ± 0.29 **(↓)**	10.28 ± 1.61 **(↑)**
S372L	3.62 ± 0.14 (↔)	5.55 ± 0.84 (↔)	11.87 ± 0.59 (↔)	2.96 ± 0.67 (↔)
G376S	3.49 ± 0.20 (↔)	6.96 ± 0.82 (↔)	12.28 ± 0.67 (↔)	3.83 ± 0.97 (↔)
G377S	nd	nd	11.68 ± 0.62 (↔)	3.36 ± 0.48 (↔)
A383S	3.58 ± 0.27 (↔)	6.42 ± 0.58 (↔)	12.20 ± 0.54 (↔)	3.88 ± 0.95 (↔)
G386R	3.48 ± 0.29 (↔)	6.55 ± 0.51 (↔)	12.13 ± 0.52 (↔)	2.89 ± 0.43 (↔)
G393S	nd	nd	11.14 ± 0.02 (↔)	2.30 ± 0.78 **(↓ ?)**
Y405C	nd	nd	12.05 ± 0.27 (↔)	5.11 ± 0.68 **(↑)**
H409L	nd	nd	11.75 ± 0.62 (↔)	2.97 ± 0.51 (↔)
G430E	nd	nd	12.22 ± 0.91 (↔)	3.71 ± 0.46 (↔)
wt	3.90 ± 0.20	5.62 ± 0.39	12.10 ± 0.81	2.85 ± 0.53
Reporter only	1	1	1	1
Reporter + RelA	1.08 ± 0.07	15.70 ± 0.22	1.12 ± 0.04	13.59 ± 1.54
non-transfected	1.32 ± 0.19	0.41 ± 0.03	1.53 ± 0.07	0.46 ± 0.04

Arrows indicate normal (↔), decreased (↓) or increased (↑) compared to the wildtype (wt) controls.

Supplementary Figure 1

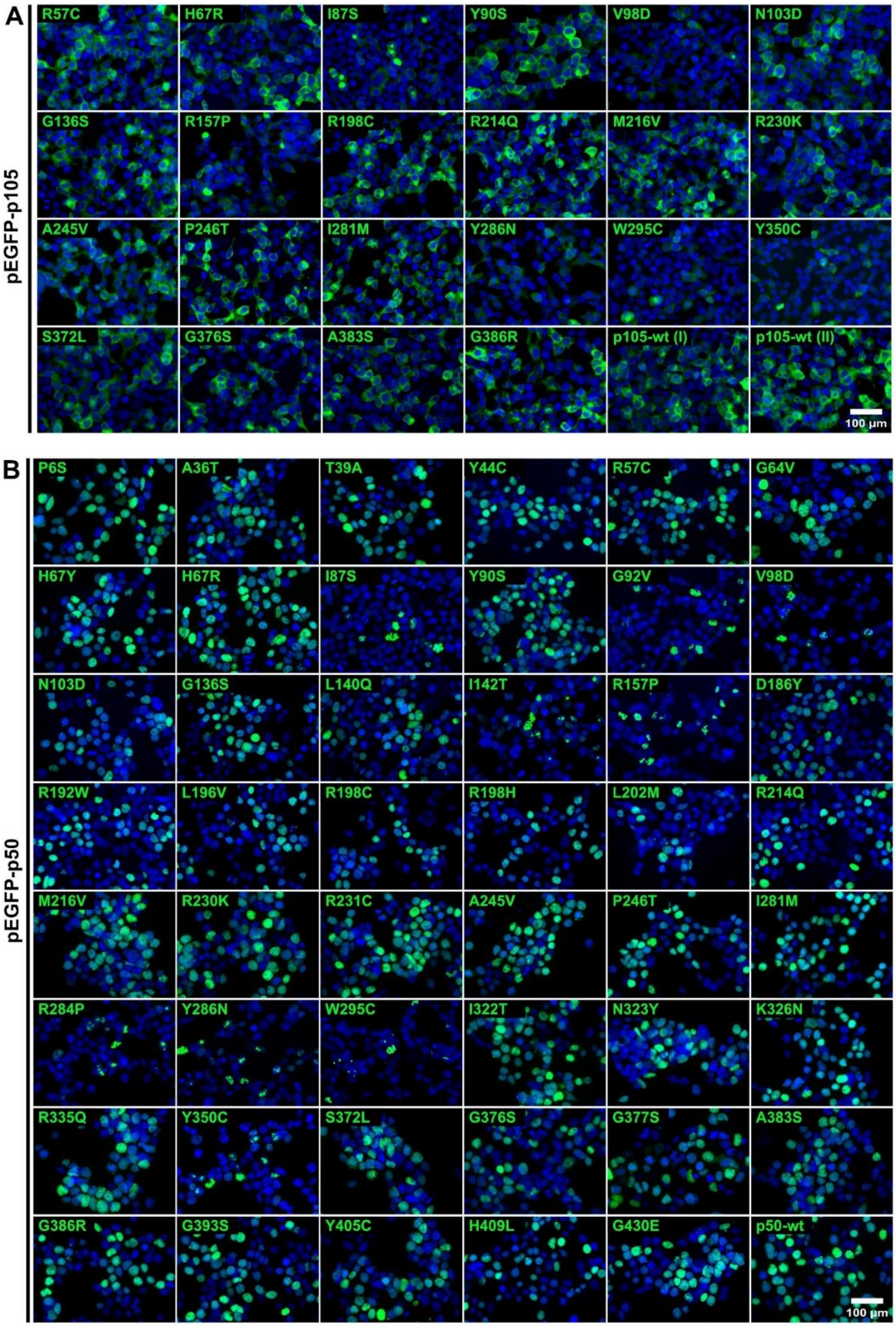

Supplementary Figure 1. Protein-decaying NFKB1 variants are indicated by limited p105 expression and sub-nuclear mis-localization of p50.

Images as shown in Figure 2A and 2B of the main manuscript. Overlay images of HEK293T cells transiently transfected with expression vectors encoding EGFP-tagged proteins (green) of wildtype or mutant p105 (A) or p50 (B). Nuclei were stained with Hoechst33342 (blue).

Supplementary Figure 2

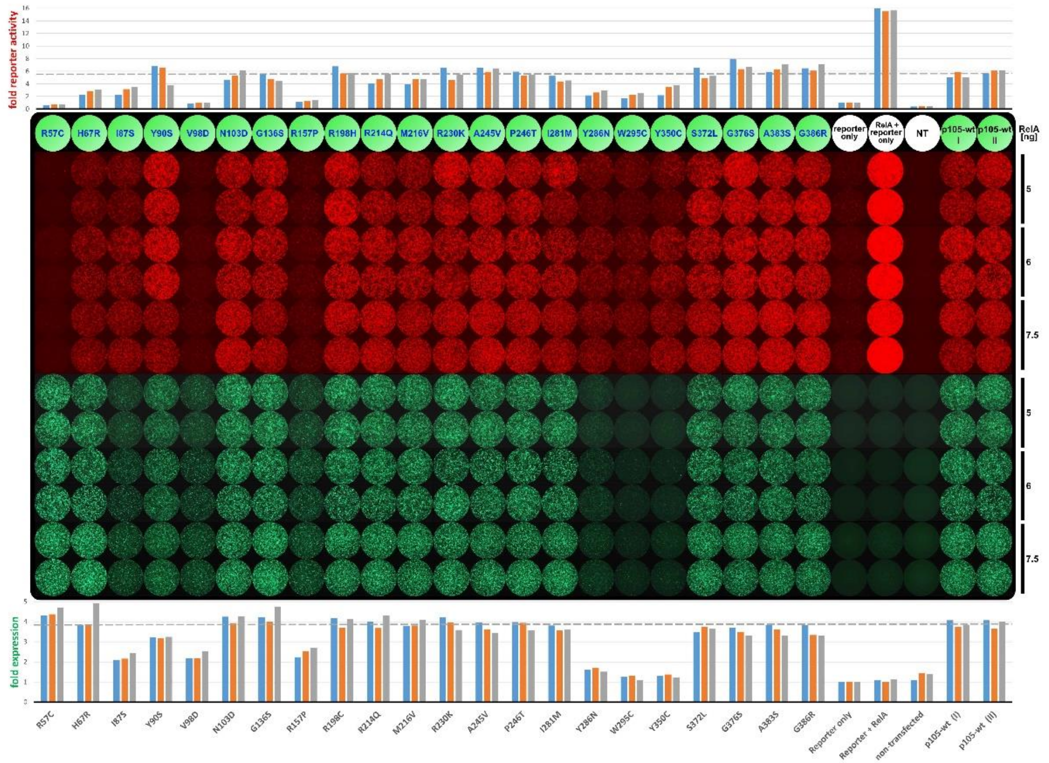

Supplementary Figure 2. RelA-dependent NF-κB reporter competition assays with p105 constructs in transiently transfected HEK293T cells Three independent experiments are shown, with duplicate transfections each. The complete panel of the experiment shown in Figure 5A is depicted, with reporter activity (red) and p50 expression (green). RelA amounts were: 5ng (top rows each), 6ng (middle rows) or 7.5ng (bottom rows). Mean intensities were calculated as an x-fold of background values (with the non-transfected samples set as 1) and are indicated by bar graphs, separately for each experiment. Average fold values of reporter activities and p105 expression are given in Supplementary Table 2. Please note the ductile effect of some variants on the reporter activity with increasing RelA.

Supplementary Figure 3

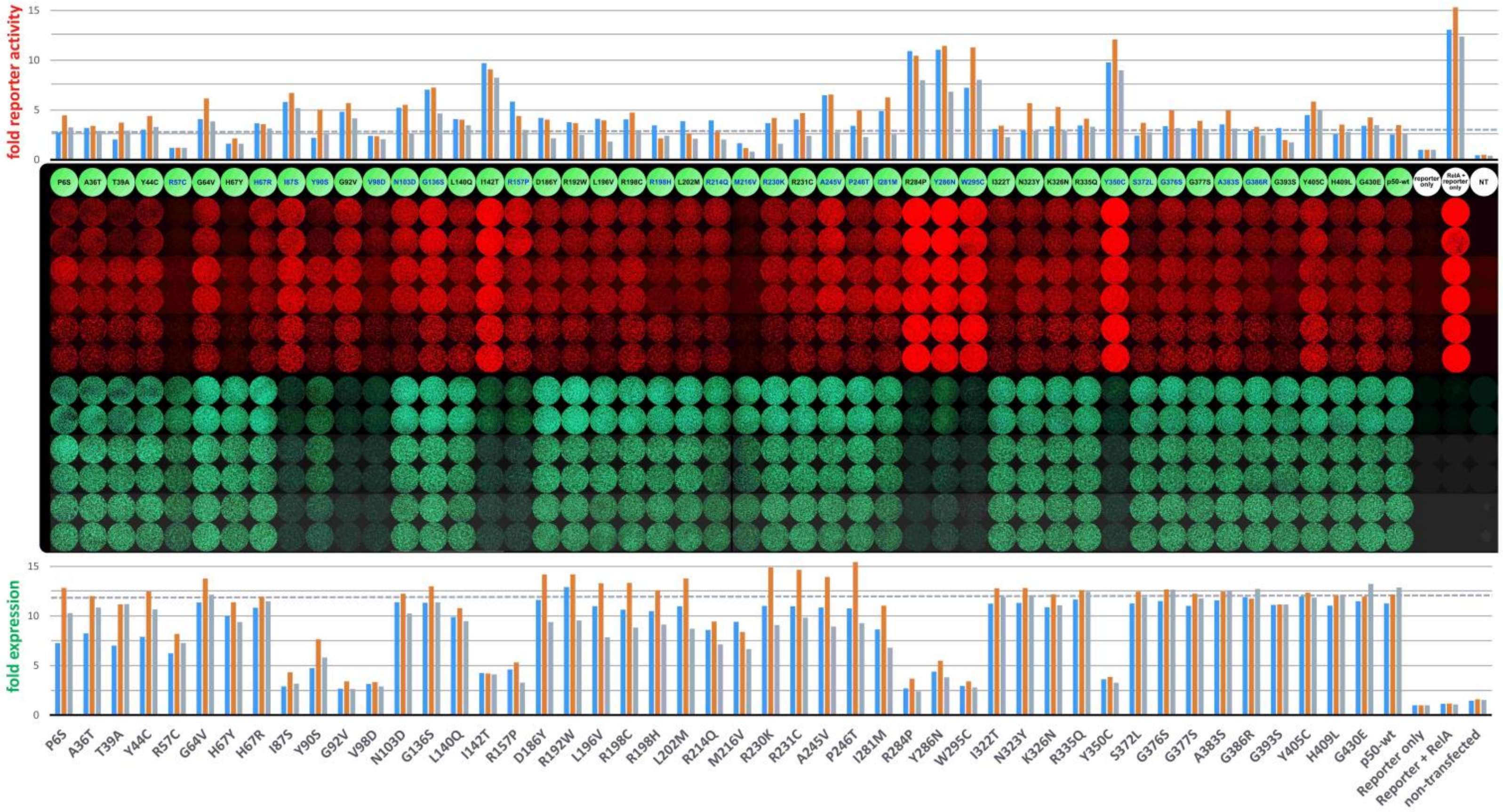

Supplementary Figure 3. Reporter Assays with transfected p50 constructs
Three independent experiments are shown, with duplicate transfections each. The complete panel of the experiments shown in Figure 5B is depicted, with reporter activity (red) and p50 expression (green). RelA amount: 5ng each. Mean intensities were calculated as an x-fold of background values (with the non-transfected samples set as 1) and are indicated by bar graphs, separately for each experiment. Average fold values of reporter activities and p50 expression are given in Supplementary Table 2.

Supplementary Table 3: Patients enrolled in this study

cDNA change	Protein change (HGVS)	Protein change (single letter code)	Sex	Family	YOB	Alias	case report	clinical synopsis	gnomAD allele count	functional defect (synopsis; see main text)	p105 and/or p50 in patient-derived cells (Western Blot)	Reference
c.16C>T	p.Pro6Ser	P6S	n.a.	—	n.a.	—	no	n.a	0	no	n.a.	Tuijnenburg et al., 2018
c.106G>A	p.Ala36Thr	A36T	M	—	2005	BM.I.1	no	CVID	0	no	n.a.	Lorenzini et al., 2020
c.115A>G	p.Thr39Ala	T39A	n.a.	—	n.a.	—	no	n.a.	11 (het)	no	n.a.	Tuijnenburg et al., 2018; Li et al., 2021
c.131A>G	p.Tyr44Cys	Y44C	n.a.	—	n.a.	—	no	n.a.	0	no	n.a.	Tuijnenburg et al., 2018
c.169C>T	p.Arg57Cys	R57C	M	—	2000	BK.II.1	yes	CVID	0	yes	n.a.	Lorenzini et al., 2020; Rojas-Restrepo et al., 2021
c.191G>T	p.Gly64Val	G64V	F	grand mother	1947	—	yes	unaffected	0	yes	n.a.	Lorenzini et al., 2020
			F	mother	1976	—	yes	CVID				
			M	son	2004	—	(yes)	unaffected				
c.199C>T	p.His67Tyr	H67Y	F	mother	1981	BL.I.1	yes	autoimmunity and immune dysregulation	0	yes/unclear	n.a.	Lorenzini et al., 2020
			F	daughter	2008	BL.II.1	yes	antibody deficiency				
c.200A>G	p.His67Arg	H67R	M	brother of II.4	(† at age 39)	F1.II.1	published	autoinflammatory disorder	0	yes (**)	normal	Kaustio et al., 2017; Lorenzini et al., 2020
			F	sister of II.1	1962	F1.II.4	published	CVID				
			F	daughter of II.1	1977	F1.III.2	published	antibody deficiency				
			M	son of II.1	1981	F1.III.3	published	CVID				
			F	daughter of II.1	1987	F1.III.6	published	CVID				
			F	daughter of II.4	1989	F1.III.7	published	autoinflammatory disorder				
			F	daughter of II.4	1992	F1.III.8	published	CVID/ Behçet's disease				
			M	son of III.2	2007	F1.IV.1	published	antibody deficiency				
			F	daughter of III.2	2010	F1.IV.2	published	antibody deficiency				
			M	father	1964	—	yes	antibody deficiency, CLL				this study
			F	daughter	2000	—	yes	antibody deficiency				
c.260T>G	p.Ile87Ser	I87S	F	—	1980 (†)	CaseG / G.II.1	yes	CVID	0	yes	reduced	Tuijnenburg et al., 2018; Lorenzini et al., 2020
c.269A>C	p.Tyr90Ser	Y90S	M	—	1956	BN.I.1	yes	CVID	0	yes/unclear	n.a.	Lorenzini et al., 2020; Rojas-Restrepo et al., 2021
c.275G>T	p.Gly92Val	G92V	F	—	2010	—	yes	antibody deficiency	0	yes	n.a.	this study
c.293T>A	p.Val98Asp	V98D	F	—	1946	CaseF / F.II.1	yes	CVID	0 (altern 1)	yes	reduced	Tuijnenburg et al., 2018; Lorenzini et al., 2020
c.307A>G	p.Asn103Asp	N103D	M	—	1979	—	yes	antibody deficiency	0	no/unclear	n.a.	this study
c.406G>A	p.Gly136Ser	G136S	n.a.	—	n.a.	—	no	n.a.	19 (het)	no	n.a.	Tuijnenburg et al., 2018; Li et al., 2021
			F	—	1951	—	no	antibody deficiency				this study
c.419T>A	p.Leu140Gln	L140Q	n.a.	—	n.a.	—	published	somatic; speckled leukoplakia	0	no	n.a.	Govindarajan et al., 2016
c.425T>C	p.Ile142Thr	I142T	M	—	2017	—	published	cytopenia, splenomegaly, lymphadenopathy	0	yes	n.a.	Duan & Feanny 2019
c.470G>C	p.Arg157Pro	R157P	F	mother of S8 / S9	n.a.	—	(yes)	Unaffected	0	yes	reduced	Schröder et al., 2019; Lorenzini et al., 2020
			M	father	1972	S8 / BO.I.2	published	CVID				
			M	brother of S8	1977	S9 / BO.I.1	published	CVID				
			F	daughter of S8	2006	—	yes	unaffected				this study
c.556G>T	p.Asp186Tyr	D186Y	F	mother	n.a.	BQ.I.1	no	autoinflammatory disorder	1 (het)	no	n.a.	Lorenzini et al., 2020
			F	daughter	n.a.	BQ.II.1	no	autoinflammatory disorder				
c.574C>T	p.Arg192Trp	R192W	n.a.	—	n.a.	—	no	n.a.	0 (altern 1)	no/unclear	n.a.	Tuijnenburg et al., 2018
c.586C>G	p.Leu196Val	L196V	M	—	1998	—	yes	CVID/CID	0	no	n.a.	this study
c.592C>T	p.Arg198Cys	R198C	M	—	1982	—	yes	CVID	2 (het)	no/unclear	n.a.	Lorenzini et al., 2020
c.593G>A	p.Arg198His	R198H	n.a.	—	n.a.	—	no	n.a.	109 (het)	no	n.a.	Tuijnenburg et al., 2018; Li et al., 2021
			n.a.	—	n.a.	—	no	n.a.				
c.604C>A	p.Leu202Met	L202M	n.a.	—	n.a.	—	no	n.a.	1 (het)	no	n.a	Tuijnenburg et al., 2018
c.641G>A	p.Arg214Gln	R214Q	M	father	1948	—	(yes)	unaffected	0 (altern 2)	yes/unclear	n.a.	Lorenzini et al., 2020; Rojas-Restrepo et al., 2021
			M	brother of BR.I.1	1971	—	(yes)	unaffected				
			M	son	1992	BR.I.1	(yes)	CVID				
c.646A>G	p.Met216Val	M216V	M	father	1956	BS.I.1	(yes)	unaffected	0 (altern 2)	yes/unclear	n.a.	Lorenzini et al., 2020; Rojas-Restrepo et al., 2021
			M	son	2011	BS.II.1	yes	antibody deficiency				
c.689G>A	p.Arg230Lys	R230K	F	—	n.a.	BT.I.1	no	antibody deficiency	0	no	n.a.	Lorenzini et al., 2020
			M	son	2006	BT.II.1	no	CVID				
			F	daughter	2012	BT.II.2	(yes)	antibody deficiency				
			F	daughter	2010	BT.II.3	(yes)	antibody deficiency				
c.691C>T	p.Arg231Cys	R231C	M	father	1974	P1	published	unaffected	14 (het) (altern 3; 11)	no	reduced	Anim et al., 2021
			M	son	1998	P2	published	CID				
			M	son	2000	P3	published	CVID				
c.734C>T	p.Ala245Val	A245V	M	—	1950	BU.I.1	yes	CVID	0 (altern 1)	yes/unclear	n.a.	Lorenzini et al., 2020
c.736C>A	p.Pro246Thr	P246T	F	—	1960	BV.I.1	no	CVID	0	no	n.a.	Lorenzini et al., 2020
c.843C>G	p.Ile281Met	I281M	M	—	1992	CaseE / E.II.1	yes	CVID	0	yes/unclear	reduced	Tuijnenburg et al., 2018; Lorenzini et al., 2020
c.851G>C	p.Arg284Pro	R284P	M	son	2003	—	yes	CVID	0	yes	n.a.	this study
			M	father	n.a	—	no	n.a.				
c.856T>A	p.Tyr286Asn	Y286N	F	—	1975	BX.I.1	yes	CVID	0	yes	n.a.	Lorenzini et al., 2020
c.885G>C	p.Trp295Cys	W295C	M	—	1956 († at age 63)	BY.I.1	yes	CVID	0	yes	n.a.	Lorenzini et al., 2020
c.965T>C	p.Ile322Thr	I322T	n.a.	—	n.a.	—	no	n.a.	3 (het) (altern 2)	no	n.a.	Tuijnenburg et al., 2018
c.967A>T	p.Asn323Tyr	N323Y	F	—	1999	P2 (*)	published	CVID (*)	0	no	n.a.	Christiansen et al., 2020
c.978A>C	p.Lys326Asn	K326N	M	—	1992	BZ.I.1	no	CVID	1459 (het)	no	n.a.	Lorenzini et al., 2020; Li et al., 2021
			M	—	1999 († at age 13)	CA.I.1	no	antibody deficiency				
c.1004G>A	p.Arg335Gln	R335Q	M	—	1991	—	no	n.a.	49 (het)	no	n.a.	Lorenzini et al., 2020
c.1049A>G	p.Tyr350Cys	Y350C	F	mother of Pat2	1945	FamA-Pat1	published	CVID	0	yes	reduced	Lorenzini et al., 2020; Fliegauf et al., 2021
			F	mother of Pat3	1976	FamA-Pat2	published	CVID				
			M	son of Pat2	2012	FamA-Pat3	published	CVID				
			M	cousin of Pat2	1973	FamA-Pat4	published	CVID				
c.1115C>T	p.Ser372Leu	S372L	M	brother	1992	—	no	CVID	1 (het)	no	n.a.	Lorenzini et al., 2020
			M	brother	1993	—	(no)	unaffected				
c.1126G>A	p.Gly376Ser	G376S	M	father	1939	—	(no)	unaffected	12 (het)	no	n.a.	Lorenzini et al., 2020
			F	daughter	1968	—	no	CVID				
			M	son	1970	—	(no)	unaffected				
c.1129G>A	p.Gly377Ser	G377S	n.a.	—	n.a.	—	no	n.a.	5 (het)	no	n.a.	Tuijnenburg et al., 2018; Li et al., 2021
c.1147G>T	p.Ala383Ser	A383S	M	—	1992	—	no	n.a.	0	no	n.a.	Lorenzini et al., 2020
c.1156G>A	p.Gly386Arg	G386R	F	—	2006	CB.I.1	yes	CVID	5 (het)	no/unclear	n.a.	Lorenzini et al., 2020; Li et al., 2021
			n.a.	—	n.a.	—	no	n.a.				Tuijnenburg et al., 2018;
			n.a.	—	n.a.	—	no	n.a.				
c.1177G>A	p.Gly393Ser	G393S	M	—	n.a.	—	no	n.a.	3 (het)	no	n.a.	Lorenzini et al., 2020
c.1214A>G	p.Tyr405Cys	Y405C	F	—	2002	—	yes	lupus, chronic thrombocytopenia	3 (het)	no	n.a.	this study
c.1226A>T	p.His409Leu	H409L	n.a.	—	n.a.	—	no	n.a.	1 (het)	no	n.a.	Tuijnenburg et al., 2018
c.1289G>A	p.Gly430Glu	G430E	F	—	1972	—	published	somatic; cervical cancer	0	no	n.a.	Yang et al., 2017

(*) P2 carries a second deleterious variant (p.Pro317_Ile322delinsLeu) on the same allele; (**) this variant has previously been described as pathogenic; n.a., data not available; (†), deceased; altern, alternative variants at the same amino acid position; YOB, year-of-birth; HGVS, human genome variation society; CVID, common variable immunodeficiency.

Case Reports

c.169C>T; exon 5; p.Arg57Cys

R57C/1. This patient was born in 2000 and presented with recurrent bolus events at the age of 12 years, when he tried to swallow pieces of meat. An esophagogastroduodenoscopy revealed eosinophilic esophagitis with quite severe strictures. In the past history the mother reported her son was prone to infections of the upper and lower respiratory tract and suffered from severe food allergic reactions. He had been instructed on how to use an epinephrine-pen after a life-threatening systemic reaction related to seafood. Unfortunately, at that time no further immunological investigations had been done. His eosinophilic esophagitis was treated with topical budesonide and strictures expanded by repeated transesophageal bougienage.

Laboratory tests in the primary immunodeficiency clinic showed blood eosinophilia in the absence of elevated or specific reactive IgE in serum, severe hypogammaglobulinemia (IgG 3.7 g/l, IgA <0.5 g/l, IgM <0.25 g/l, and IgE 26 IU/l) expending over all IgG subclasses (IgG1 2.4 g/l, IgG2 0.4 g/l, IgG3 0.2 g/l, and IgG4 0.04 g/l) and complete absence of any specific immune responses to standard vaccinations, still six weeks after re-challenging. Lymphocyte phenotyping was compatible with CVID, EURO Class B+smB+TrnormCD21norm. No evidence of protein loss in urine or stool sampling was found. Genetic panel-testing of known PID genes revealed a new mutation in *NFKB1*.

Initiation of IgG replacement therapy was challenging, due to moderate systemic allergic reactions to different immunoglobulin products even when adequate premedication (glucocorticoids and antihistamines) was used. Fortunately, after the invention of subcutaneous IgG substitution all allergic reactions subsided completely and no infections occurred ever since. Problems with swallowing and histologic eosinophilic esophagitis recurred after termination of budesonide therapy and seemed not to be beneficially influenced by IgG replacement. Therefore, the patient is on long-term topical budesonide treatment and otherwise in good general condition.

c.191G>T; exon 5; p.Gly64Val

G64V/2. This female patient was born to non-consanguineous parents in 1976, and had suffered since childhood from recurrent upper and lower respiratory tract infections often needing antibiotics, and had been diagnosed with allergic asthma. At 34 years, she was admitted with a severe pneumonia, and further diagnostic testing was done. Her blood chemistry showed low IgG (5.6 g/L), low IgA (0.6 g/L) and normal IgM (0.96 g/L) levels. The overall antibody response to vaccination with Pneumovax® was adequate, however antibody responses against separate pneumococcal serotypes were not assessed. After further immunological evaluation, she was diagnosed with CVID EuroClass SmB+CD21norm and immunoglobulin replacement therapy was started. Since then, the frequency and severity of her infections has markedly decreased, with only a limited need for antibiotics. Thus far, she has not developed bronchiectasis or chronic lung disease. However, she suffers from severe fatigue and neuro-psychiatric symptoms. At the age of 39, she had bilateral optic neuritis/papillitis causing loss of vision and eye pain. She was treated with corticosteroids with good response, although her vision was only partially restored. Extensive neurological work-up remained negative; multiple sclerosis was excluded. Corticosteroids could be successfully weaned, and no other immunosuppressive drugs were started. In the next years, she presented with several episodes of weakness and paresthesia in the upper or lower limbs, each time with normal MRI findings and spontaneous recovery. These episodes were considered to be triggered by severe psychological stress.

At the age of 40, genetic testing revealed the heterozygous mutation in *NFKB1*, regarded as likely pathogenic by all *in silico* prediction tools. The same *NFKB1* mutation was also detected in her mother as well as in one of her sons, confirming an autosomal dominant inheritance across three generations.

G64V/1. This female patient was born in 1947 and is the mother of patient G64V/2. She had breast cancer at the age of 64, which is currently in remission and she is in good general condition. The *NFKB1* mutation was detected at age 68. She was found to have very low IgG levels (3.7 g/dL), normal IgA (2.2 g/L) and IgM (1.0 g/L) levels, and inadequate antibody response to vaccination with Pneumovax® (serotype analysis). B cell phenotyping was compatible with EuroClass SmB+CD21norm. Unlike her daughter, she does not have a history of recurrent or severe infections or immune dysregulation. She does not receive immunoglobulin replacement therapy.

G64V/3. The son of patient G64V/2 was born in 2004. At age 13 the same *NFKB1* mutation was detected. Currently he has no clinical or immunological phenotype of a humoral immunodeficiency (normal immunoglobulins, vaccine responses and B cell phenotyping).

<u>c.199C>T; exon 5; p.His67Tyr (protein defect unclear)</u>

H67Y/2. This female patient was born in 2008 to unrelated parents. The patient presented at 6 years old with tender cervical lymphadenopathy that was nonresponsive to antibiotics targeting *Staphylococcus* and *Streptococcus*. Biopsy showing necrotizing granulomatous inflammation and culture was positive for *Mycobacterium avium intracellulare* complex (MAC). Dual antibiotic therapy was initiated but the infection persisted and several months later the patient developed severe abdominal pain. Imaging revealed mesenteric and retroperitoneal lymphadenopathy, and blood and stool cultures were positive for MAC. The antibiotic regimen was broadened and she clinically improved, but blood cultures remained positive. At 8 years, referral was made for immunologic evaluation which revealed low memory and class-switched memory B lymphocytes, and poor vaccine responses. Whole exome sequencing revealed the H67Y variant in *NFKB1*. Genetic defects associated with Mendelian susceptibility to mycobacterial disease (MSMD) were not found. Until the presentation with disseminated MAC, she was relatively healthy, with about 3 ear infections, no episodes of pneumonia, and a limited episode of thrush during infancy. The patient was treated with immunoglobulin replacement therapy, and combination MAC antibiotics with adjuvant interferon-gamma, but she remained with positive mycobacterial cultures. Therefore, at age 8 she received a haplo-identical hematopoietic stem cell transplantation (reported in Dimitrova et al, Biol Blood Marrow Transplant 2021). The transplant was complicated by graft rejection, but with clearance of the Mycobacterial infection. The patient is currently 13 years old, has not had return of the Mycobacterial infection, and has remained well on azithromycin and immunoglobulin replacement therapy.

H67Y/1. This patient was born in 1981 and is the mother of patient H67Y/2. During the evaluation of her daughter, family history revealed recurrent infections and autoimmunity in the mother leading to her evaluation as a patient. She presented at age 18 years with hypothyroidism followed by insulin dependent diabetes in her early 30s. She developed pernicious anemia in her early 20s with a recurrence in her mid-30s. She does not have a history of childhood infections, but has had frequent sinus infections starting in early adulthood, had sinus surgery in her early 30s, and has had two pneumonias as an adult. She also had frequent vaginal yeast infections and onychomycosis. Immunologic evaluation revealed low serum IgA at 2g/L and IgG at 4.02 g/L, and low vaccine antibody responses. T and B lymphocyte numbers were normal, but memory class-switched B cells were low. She was initiated on immunoglobulin replacement therapy with improvement in the frequency of

her infections, fluconazole that resolved the onychomycosis and vaginal yeast infections, and replacement of insulin, thyroid hormone and vitamin B12.

c.200A>G; exon 5; p.His67Arg

H67R/1 - H67R/9. Case reports of nine members of this Finnish family (F1) were described previously (Kaustio et al., 2017). Western blot analysis of PBMCs derived from F1.III-8 showed p105/p50 levels comparable to healthy control samples (Kaustio et al., 2017).

H67R/10. The father of patient H67R/11 was born in 1964. He started with recurrent respiratory tract infections at age 27 and suffered from severe aphthous ulcers. He carries a diagnosis of chronic lymphocytic leukemia and antibody deficiency and has been on immunoglobulin replacement therapy for over 20 years.

H67R/11. This female patient (daughter of H67R/10) was born in 2000. She presented at 14 years of age with a history of recurrent upper and lower respiratory tract infections. Since five years of age, she suffered with chronic fatigue that was worse when she had an active infection. Occasional aphthous ulcers responded to topical steroid in dental paste. Local laboratory studies revealed normal routine blood counts, chemistry panels, sedimentation rates and CRP measurements. Serum IgG was low at 4,58 g/L; IgA (1,18 g/L) and (IgM 0,99 g/L) were within reference ranges for age. At 15 years of age, she responded well to *Haemophilus influenzae* type b immunization (>9.00 µg/mL) and Pneumovax® vaccine (positive for 17 of 23 serotypes tested). Lymphocyte subsets showed normal CD3+, CD3+CD4+, CD3+CD8+, CD19+ and CD16+CD56+ cells and switched memory B-cells. Epstein-Barr virus whole blood quantitative PCR was positive (4200 cc/ml) and has remained detectable over the past several years. Epstein-Barr virus antibody panel demonstrated persistently negative Epstein-Barr virus nuclear antigen antibody. Triad exome sequencing at the Children's Hospital Los Angeles Center for Personalized Medicine Clinical Genomics laboratory identified a heterozygous variant in *NFKB1* in the patient and her father that was predicted to be ”likely pathogenic”.

c.260T>G; exon 6; p.Ile87Ser

I87S/1. This female patient (Case G; Tuinenburg et al., 2018) was born in 1980 and was one of three siblings born to non-consanguineous parents. She had an uneventful childhood and received a full complement of childhood vaccinations as per immunisation schedule. She presented at age 18 with acute respiratory distress syndrome secondary to pneumonia requiring ventilation and a ten day intensive care admission. This episode was preceded by a tongue piercing though it was unknown if the two were related. She continued to suffer with predominantly lower respiratory tract infections requiring multiple hospital admissions. Aged 21 years old, immunological evaluation was undertaken. Pan-hypogammaglobulinemia was noted and she was subsequently diagnosed with CVID. Lymphocyte phenotyping revealed CD19 lymphopenia with reduced percentage of switched memory B cells, fitting into the category of CVID EUROClass $B^{+}smB^{-}CD21^{norm}$. Immunoglobulin replacement was initiated with prophylactic antibiotic cover. The patient had a 6-10 pack year history on presentation and smoking cessation was recommended. Bronchiectasis and possible emphysema were noted on CT chest imaging with reduced gas transfer on pulmonary function testing, TLCO 61% predicted and KCO 88% predicted. She gave birth to a healthy daughter the year after. At age 26 she had a prolonged intensive care admission for pneumonia requiring ventilation and a tracheostomy. She had a further admission with profuse diarrhea with *Clostridium difficile* infection. Upper and lower gastrointestinal endoscopy showed inflammation in the upper and lower duodenum with marked increase in intraepithelial lymphocytes and a mild increase in inflammatory cells in the lamina propria, gastric ulceration and colonic

inflammatory changes. An ultrasound of the upper abdomen showed a mildly enlarged spleen and an oedematous ascending colon and caecum. With incremental immunoglobulin doses, her IgG level eventually remained at a therapeutic level. An expansion of CD21low B cells was added to the previous laboratory findings. Her clinical course thereafter consisted of progressive exertional dyspnoea and wheeze, productive morning cough and loose bowel motions. The patient continued to smoke actively until age 27. Both *Haemophilus influenzae* and *Pseudomonas aeruginosa* were isolated from sputum microscopy and culture on numerous occasions, requiring intravenous and/or oral antibiotic therapy. Aged 29 she had a right lower lobectomy for worsening bronchiectasis though she continued to suffer with recurrent respiratory tract infections. Percentage of CD19 B cells was under 1%, being reclassified as EUROClass B. At age 31, she underwent a liver biopsy with features consistent with nodular regenerative hyperplasia. At age 32, she had a portacath inserted for poor venous access. Aged 33 years old, she twice isolated *Mycobacterium avium intracellulare* on sputum microscopy and culture. Mycobacterial treatment was initiated though this was poorly tolerated in the form of combination treatment. This was eventually stopped. Pulmonary function tests continued to decline with severe bronchiectasis and dyspnea and she was assessed for a potential lung transplant. Repeated liver biopsy performed for deteriorating liver function tests and for suitability of transplant was essentially nonspecific. At age 35 she commenced home oxygen therapy. Repeated surveillance of mycobacterial sputum cultures was negative. Her CVID enteropathy, with no evidence of malabsorption, remained problematic with symptomatic improvement with oral steroid courses. She required enteral nutrition with insertion of a radiologically inserted gastrostomy (RIG) tube for poor weight gain. She was deemed unsuitable for lung transplant and consequently a hematopoietic stem cell transplant was also excluded. Over the last two years of life, her oxygen requirements increased and pulmonary function tests worsened. Her weight improved with RIG feeding. At age 37 she was admitted for intravenous antibiotics and aminoglycoside nebulizers for a *Pseudomonas* infection. Several months later, she presented to her local hospital with bloody diarrhea, up to 10 episodes a day. Stool samples were negative for enteropathogenic bacteria, viruses, parasites and *Clostridium difficile* toxin. Abdominal plain film showed some bowel thickening but no megacolon. Colonic biopsies were consistent with colitis and fecal calprotectin levels were markedly increased. Her symptoms remained refractory to high dose steroids and she underwent bowel surgery. She passed away secondary to postoperative complications. Western blotting showed reduced expression of p50 in patient-derived neutrophils (Tuijnenburg et al., 2018).

c.269A>C; exon 6; p.Tyr90Ser

Y90S/1. This male patient was born to unrelated parents in 1956. He suffered from psoriasis and respiratory infections including chronic sinusitis and two pneumonias at ages 25 and 30. The first pneumonia was complicated by abscess formation in the left lung, which required thoracic surgery. Following surgery, the patient was infected with hepatitis C virus from a blood transfusion. At age 30 he underwent surgery of the paranasal sinuses due to the chronic sinusitis. Two years later a tonsillectomy was performed. In his 30's, the patient developed liver cirrhosis, portal hypertension and splenomegaly. He also suffered from erosive gastritis. At age 39 hypogammaglobulinemia was incidentally detected (IgG 3.5 g/l, IgA 0.3 g/l and IgM 0.2 g/l). After further immunological evaluation, he was diagnosed with CVID EUROClass B^+ $CD21^{low}$ smB^+ Tr ^{norm}. Since then he has been undergoing immunoglobulin replacement therapy. Evaluation of his chronic gastritis and duodenitis at age 50 revealed *Helicobacter pylori*, which was successfully treated with triple therapy. At age 51, he had grade III esophageal varices and variceal bleeding. After several ligations were performed, he had grade I residual esophageal varices. At age 59 hepatitis C was cured after antiviral therapy.

In addition, at age 61, he suffered from a portal vein thrombosis and a transjugular intrahepatic portosystemic shunt was performed. Currently, he is in good general condition, without infections that need antibiotic therapy. He has thrombocytopenia, possibly due to his splenomegaly and B cell lymphopenia with low number of marginal zone B cells, class switched memory B cells and plasmablasts.

c.275G>T; exon 6; p.Gly92Val

G92V/1. This patient of Belarusian origin was born to unrelated parents in 2010. At the age of 8 years severe anemia (Hb 26 g/L) was the reason for hospitalization in the intensive care unit, she received treatment with hemotransfusion, dexamethasone, intravenous immunoglobulin and plasmapheresis. After 4 weeks of intensive care hospitalization thrombosis of the right external and common iliac vein was diagnosed and fragmin was added to the therapy. Additionally computer tomography revealed fibrotic changes in both lungs, hepatosplenomegaly and right kidney stones. At the age of 8 years and 6 months the diagnosis of acute gangrenous cholecystitis was established and she underwent cholecystectomy. First immunologic investigation showed low number of recent thymic emigrants (CD4+CD45RA+CD31+ 9.74%, 73 cells/µl), switched memory B cells (CD19+CD27+IgD- 2%) and increased CD21low and CD21lowCD38low B cells accompanying with low concentration of IgG 2.5 g/L. Since then she receives antibody replacement therapy.

c.293T>A; exon 6; p.Val98Asp

V98D/1. This female patient (CaseF; Tuijnenburg et al., 2018) was born in 1946 and was one of four siblings. She had suffered with recurrent sinopulmonary infections for a number of years and had a chronic cough expectorating yellow colored phlegm. Aged 48 years old, she was diagnosed with CVID and commenced on Immunoglobulin replacement therapy. Bronchiectasis was noted on chest imaging. At age 50 she reported recurrent palpitations and was noted to have a systolic murmur. An echocardiogram and 24 hour tape were essentially normal. She remained clinically stable on both immunoglobulin treatment and prophylactic antibiotics with several sinopulmonary infections a year. At age 54, she was noted to have a progressive decline in gas transfer; TLCO 45% predicted and KCO 82% predicted compared with values of 83% for both, three years earlier. Alkaline phosphatase levels were noted to be rising. Chest plain film showed changes consistent with bilateral pulmonary fibrosis and bronchiectasis. She was treated for granulomatous CVID lung disease and responded well to oral steroid treatment with improvement in gas transfer. At age 58, she was admitted for investigation of a central abdominal mass, drenching night sweats and 13 kg weight loss over a three-month period. CT abdominal imaging showed a retroperitoneal mass of approximately 20 x 20 x 10 cm, which on biopsy was confirmed to be a stage 2 B follicular lymphoma with no bone marrow involvement. She completed 6 cycles of chlorambucil. She continued to have some subtle abdominal symptoms and drenching night sweats, and a repeat staging CT the year after showed a new finding of two soft tissue masses medial and anteromedial to the lower pole of the left kidney. She received further Chlorambucil for 14 days with resolution of the left peri-renal masses but no interval change of a suprarenal mass. A ‘watch and wait’ approach was taken. She was investigated for palpitations and was found to have mild pulmonary hypertension on an echocardiogram with good left ventricular function. At age 59 lymphocyte phenotyping was compatible with CVID, EURO Class $B^{+}smB^{-}CD21^{lo}$. At age 62, a new soft tissue mass lateral to the right kidney and a new soft tissue mass arising from the posterior medical aspect of the left diaphragm, suggestive of relapse of the lymphoma were noted on a CT scan performed for back pain with no B symptoms, weight loss or

lymphadenopathy. She completed six courses of CVP-R treatment (vincristine, cyclophosphamide, prednisolone and rituximab) and continued on three monthly rituximab for a further two years. By age 64 the pulmonary hypertension was noted to have worsened with a pulmonary artery pressure of 40mmHg. She continued on long term low dose prednisolone for her lung disease. At age 66 she reported intermittent episodes of cellulitis affecting her right leg. At age 67 she was noted to be biochemically thyrotoxic but clinically asymptomatic with an undetectable TSH level and raised free T4 and T3. She was found to have a multinodular goitre. At age 70 she was admitted to intensive care with chest sepsis. Basal consolidation and pulmonary oedema were noted on plain chest film. *Elizabethkingia meningosepticum* was isolated from blood cultures and she was treated effectively with intravenous piperacillin/tazobactam and vancomycin. That year she also received ciprofloxacin for a *Pseudomonas* chest infection. At age 72, she presented with a 5th episode of right leg cellulitis within nine months, requiring intravenous piperacillin/tazobactam, clindamycin and flucloxacillin. Blood cultures were negative. She represented the year after confused, disorientated with diplopia, right field loss and inattention on examination. Blood cultures again isolated *Elizabethkingia meningoseptica* and she was treated with piperacillin/tazobactam. Her confusion and visual symptoms worsened. Appearances on MRI head were suggestive of widespread vasogenic oedema. Posterior reversible encephalopathy syndrome or progressive multifocal leukoencephalopathy were both considered as possible differential diagnosis. Western blot analysis of patient-derived neutrophils indicated reduced p50 expression (Tuijnenburg et al., 2018).

<u>c.425T>C; exon 7; p.Ile142Thr</u>

I142T/1. This case was reported previously (Duan and Feanny 2019).

<u>c.470G>C; exon 7; p.Arg157Pro</u>

R157P/1. This male patient was born to non-consanguineous Caucasian healthy parents of German descent in 1972 (Schröder et al., 2019). Apart from a herpes zoster infection at the age of 6 years, during childhood and adolescence no increased susceptibility to infections was apparent. At age 30 he developed myocarditis. At 41 years of age, he suffered from a prolonged perforating otitis media. Half a year later, pneumonia was diagnosed. Beginning of his increased susceptibility to infections correlates with the start of day care of his child. A mild splenomegaly was diagnosed first at 42 years. At initial presentation he had a complete agammaglobulinemia, normal absolute B cell counts and fulfilling diagnostic criteria for CVID with decreased percentages for class switched memory B cells and switched plasmablasts (EURO Class B+smB-21norm and Freiburger classification group Ib) and normal T cells and T cell subsets. Diagnosis of CVID in this patient resulted in immunological work-up of his younger brother (R157P/2). The same *NFKB1* variant was found in both brothers, their unaffected mother and his daughter (R157P/3). Western blotting demonstrated reduced p105/p50 levels in PBMCs (data not shown).

R157P/2. This male patient (Schröder et al., 2019) was born in 1977 and is the brother of patient R157P/1. He had a history of recurrent respiratory tract infections, including bronchitis, sinusitis and a severe pneumococcal pneumonia with sepsis. He reported that his three years older brother suffered from similar recurrent infections. At age 39 he underwent immunological evaluation due to the previous history. Immunological investigations revealed panhypo-gammaglobulinemia (IgG 0.45 g/l, IgA <0.26 g/l, IgM <0.18 g/l), low percentage of B and NK cells, decreased percentage of class-switched memory B cells and increased percentage of transitional B cells and plasmablasts. On the basis of the above mentioned infection record, he was diagnosed with CVID, EURO Class B^{+} smB^{-} $CD21^{norm}$ Tr^{high}. The patient was set on

immunoglobulin replacement treatment and presented since then no further severe infections. Patient derived PBMCs showed reduced p105/p50 expression (Schröder et al., 2019)

R157P/3. This female patient was born in 2006 and is the first of two children of patient R157P/1. Both parents were unrelated. The pregnancy was uneventful, apart from Caesarean section due to HELLP-syndrome of the mother. Since 2 years of age, she has been suffering from relapsing abdominal pain and diarrhea (5-7 stools per day). Gastroenterologic diagnostic was not able to detect a cause for the symptoms and she had no failure to thrive. Treatment with loperamid was commenced with improvement of the symptoms. At 2 years of age, an ophthalmological examination showed reduced marginal sharpness of the optic nerve on both sides. MRI-scan of the head and lumbar puncture including measurement of the intracranial pressure revealed no abnormalities. Except for recurrent sinusitis no invasive infections or other signs of immune dysregulation appeared. Immunological evaluation found normal immunoglobulin levels for IgG (inclusive IgG-subclasses), IgA and IgM, normal T- and B-cell-immunity and measurable vaccination levels (diphtheria, tetanus, haemophilus, pneumococcus). Molecular genetic examination revealed the same variant in *NFKB1* as in the girl's father, uncle and the mother of the father.

c.586C>G; exon 8; p.Leu196Val

L196V/1. This male patient was born in 1998. He was born to non-consanguineous parents. At birth, he received supplementary oxygen due to hypoglycemia, neonatal sepsis and hemolytic anemia (Coombs positive). During infancy he developed spasmodic cough, workup for cystic fibrosis was negative. At 1 year he presented failure to thrive. He was hospitalized at 3 years of age with severe neutropenia, suspected Evans syndrome, and cellulitis with Pseudomonas aeruginosa. Schwachman-Diamond syndrome investigations were negative. Laboratory tests showed neutropenia, hypogammaglobulinemia and low IgG1 levels. Anemia and thrombocytopenia were intermittent, antiplatelet-antibodies and positive Coombs were detected in each episode. He was treated with intravenous immunoglobulin, red cell concentrates transfusion, G-CSF plus antibiotics. CVID was diagnosed due to hypogammaglobulinemia (IgG 2.84g/l, IgM 0.38 g/l, IgA not detectable), T cell lymphopenia and no response to tetanus or pneumococcal vaccines. Immunoglobulin replacement therapy was started. Panel sequencing was negative for X-linked syndromes, type 2 and 3 Hyper IgM Syndromes. Bone marrow aspirate showed richly cellular marrow with a marked left shift. There were no morphologic abnormalities. Multilineage hematopoiesis was normal for age. He had a microcytic, hypochromic anemia with monocytosis, but no evidence of leukemia. He continued to have leukopenia and thrombocytopenia throughout his childhood. His bone age was determined to be 5 years when he was only 3 years and 7 months old (about 2 standard deviations above the chronological age). He received live viral vaccines, including VVZ. At 4 years, old he developed a vesicular lesion on the right small finger which progressively tracked up the right arm in a dermatomal fashion. He was hospitalized with a vaccine strain herpes zoster with no neurologic symptoms. Lumbar puncture showed pleocytosis but was negative for VZV. He had frequent infections including pneumonia, chronic sinusitis, epididymitis, but were later well controlled since switching to subcutaneous immunoglobulin. He was diagnosed with growth hormone deficiency and supplemented with it until 14 years of age. He developed retinopathy due to an unspecified cone dystrophy and is legally blind. At 19 years, he presented with a severe autoimmune hemolytic anemia, and was treated with prednisone and solumedrol. He later relapsed, and was treated with rituximab. WES revealed three variants of unknown significance: in *NFKB1* (c.586C>G; L196V, heterozygous), in *RIMS1* (c.3182C>A; W1061X, heterozygous) and in *TNIK* (c.1220A>T; E407V, heterozygous). At 20 years, he was hospitalized with fever, severe neutropenia, recurrent painful genital and oral ulcers. He received acyclovir. Blood CMV, EBV, HHV6, an HSV PCR were all negative. CRP was

elevated. Bone marrow biopsy due to severe neutropenia was performed showing megakaryocytic hyperplasia with dysplastic changes noted among the maturing megakaryocytes, maturing myeloid lineage cells demonstrating relative maturation arrest with extremely rare maturing granulocytes. Neupogen was given as treatment. At age 21, he was re-evaluated due to worsening T cell lymphopenia and autoimmune hemolytic anemia. A PID panel showed a heterozygous pathogenic variant in SPINK5 (c.2671C>T; R891X), and a heterozygous VUS in CARD9 (c.923_925del; L308del) and another heterozygous VUS in CHD7 (c.1496_1497delinsGC; Q499R). He developed painful recurrent herpetic lesions of feet treated with antivirals. During the pandemic he had no COVID infection, and has received both Regeneron and Evusheld. He continues to be monitored closely for autoimmune hemolytic anemia, recurrent viral infections, and is maintained on subcutaneous immunoglobulin replacement and prophylactic antibiotics s/p rituximab with continued B and T cell lymphopenia.

c.592C>T; exon 8; p.Arg198Cys (protein defect unclear)

R198C/1. This male patient was born in 1982 to non-consanguineous parents without a previous family history of immune system disorders. Vaccination history and childhood development were unremarkable. Disease onset was at age 7 with a recurrent and chronic cough. Low body temperature and feeling cold even in the warm seasons have been documented since the age of 10. The patient also had a history of recurrent upper respiratory tract infections. He had herpes zoster at age 25. At age 28, he was hospitalized because of kidney stones. At the time, the patient had submandibular and axillary lymphadenopathy, associated with enteropathy and weight loss but no hepatosplenomegaly. Endocrine, rheumatology, and hematology work-up were normal without any evidence of malignancy. Immunological analyses revealed decreased immunoglobulin levels (IgG 3.0 g/l, IgA 0.1 g/l, IgM 0.4 g/l), absence of specific antibody response (anti-tetanus <0.01 IU/ml and anti-diphtheria <0.01 IU/ml), but normal B cell counts (CD3+ 66%, CD19+ 12%), indicating the clinical diagnosis of CVID. The B cell subset analysis of this patient was compatible with CVID Freiburg Class Ia, EURO B+ smB+ $CD21^{low}$ Tr^{norm}. After intravenous immunoglobulin replacement, lymphadenopathies improved and he had a good infection control, however, the gastrointestinal manifestation did not respond to immunoglobulin treatment or other immunomodulators including corticosteroid, azathioprine, sirolimus and mesalamine.

c.641G>A; exon 8; p.Arg214Gln (protein defect unclear)

R214Q/1. This male patient was born in 1992 to a non-consanguineous couple. He has two healthy siblings. Shortly after birth he developed bronchitis and an immunodeficiency was suspected. However, he did not have any increased susceptibility to infection until he became 17. Since that age, he started having recurrent bronchitis 3 to 4 times per year, which required antibiotic therapy. He did not have other major infections nor lymphadenopathy or hepatosplenomegaly. When he was 20, he had an abscess of 1.5 cm in the right gluteus, which was successfully treated with ammonium bituminosulfonate. At age 21 he started having intermittent diarrhea. At age 22 he was immunologically evaluated. Immunoglobulin levels showed decreased immunoglobulin levels (IgG 3.9 g/l, IgA 0.22 g/l, IgM 0.23 g/l), and subclasses IgG1 and IgG2 were reduced. Response to tetanus and diphtheria vaccination was normal, whereas his pneumococcal polysaccharide response was non-protective. Lymphocyte phenotyping revealed decreased transitional and class-switched memory B cells, absent plasmablasts and increased CD21low B cells. The patient was therefore diagnosed with CVID, EURO Class $B^{+}smB^{+}CD21^{low}Tr^{norm}$. Since then, he has received antibody replacement therapy. Currently he has a good exercise tolerance and good infection control,

his only problems being low grade esophageal reflux, and intermittent diarrheal episodes without weight loss. The patient also reports skin folliculitis.

R214Q/2 and R214Q/3. The oldest brother and the father of R214Q/1 carry the same *NFKB1* variant but are completely asymptomatic.

c.646A>G; exon 8; p.Met216Val (protein defect unclear)

M216V/1. This male patient born in 2012 is the first and only child of non-consanguineous Caucasian parents. His perinatal history was unremarkable. A differential blood count at 6 months of age, which was conducted during a routine screening investigation without clinical symptoms, showed marked neutropenia with an absolute neutrophil count of <500/µl and prompted further evaluations. A bone marrow puncture showed normal maturation of all hematopoietic lineages. His previous peripheral neutropenia spontaneously resolved within the first year of life. Transient autoimmune triggered neutropenia was assumed. Pathological infections never occurred. Yet, the neutropenia triggered work-up also identified marked hypogammaglobulinemia with barely detectable IgG, IgA and IgM. Vaccine titers were also undetectable despite three previous vaccinations. Peripheral B cell numbers were normal for age and showed normal differentiation. Bruton tyrosine kinase (BTK) was detectable by flow cytometry. Reduced upregulation of CD25, OX40 and CD40L on CD4+ T cells after stimulation suggested a T cell activation defect. Subcutaneous IgG replacement was initiated as prophylactic treatment. The patient was followed closely in 6 monthly intervals. His psychomotor development was normal and he never experienced any relevant infections or signs of immune dysregulation until today. From age 4 onward he showed increasing IgG levels despite low IgG replacement for weight and IgG replacement was eventually paused at 6 years of age. Further monitoring showed regular IgG production, incl. normal vaccine responses. Yet, IgA and IgM remained below normal limits. At present the patient remains asymptomatic without any treatment.

M216V/2. This male patient was born in 1956 to a non-consanguineous couple and is the father of patient M216V/1. He suffered from bronchial asthma, coronary heart disease and compression of nerve roots due to intervertebral disc degeneration but lacked a history of susceptibility to infections or autoimmune phenomena. At age 57, he had an episode of fatigue and joint pain with elevated inflammatory markers. At age 60, the same *NFKB1* variant as in his son was identified, which triggered an immunological evaluation. Immunoglobulin levels showed decreased IgG and IgM (IgG 6.17 g/l, IgA 1.70 g/l, IgM 0.24 g/l), with a selective IgG2 reduction (0.90 g/L). Tetanus antibodies were protective, but diphtheria vaccination response was nonprotective. Lymphocyte phenotyping revealed an expansion of CD4+ T cells and a light increase in CD21low B cells. Currently, he is in a good general condition without IgG replacement.

c.691C>T; exon 8; p. p.Arg231Cys (protein defect unclear)

R231C/1 – R231C/3. Case reports of three affected family members were described previously (Anim et al., 2021). Western blot analysis of PBMCs indicated reduced p105/p50 levels in all three family members (Anim et al., 2021).

c.734C>T; exon 9; p.Ala245Val

A245V/1. This male patient was born in 1950 to unrelated parents. At age 61, he developed chronic diarrhea and lost 20 kg in the following two years. At age 63, hypogammaglobulinemia of all main immunoglobulin classes was documented (IgG 2.5 g/l, IgM 0.24 g/l, IgA <0.06 g/l). Tetanus antibodies were within normal range. Response to pneumococcal vaccine was not

tested. He had no previous history of recurrent infections. Age 63 he had two episodes of Pneumonia. Immunoglobulin replacement therapy was started due to high suspicion of CVID $B^{+}smB^{+}21^{norm}Tr^{norm}$. Mesenterial and retroperitoneal lymphoproliferation was described in abdomen CT. Bone marrow and lymph node biopsy showed no evidence of a lymphoma. Endoscopy and biopsy showed villous atrophy with mucosal mosaic pattern and proliferation of intraepithelial lymphocytes, findings comparable to celiac disease. However, both transglutaminase- and gliadin-antibodies and HLA DQ2 and DQ8 were negative. A gluten free diet was tried and later stopped due to lack of improvement. These findings were compatible with CVID enteropathy. Diarrhea improved slightly with budesonide. In addition, a Norovirus infection was diagnosed. After persisting Norovirus detection, eradication was attempted with ribavirin at age 65 with no success. A mild hepatopathy in the context of the enteropathy was diagnosed due to elevation of transaminases. Age 65 he underwent surgery due to a slipped disk with nerve compression. The following year two kidney stones were surgically removed. He developed secondary lactose and fructose intolerance and kept losing weight due to intestinal malabsorption. Age 71 a new Norovirus eradication attempt was made with nitazoxanide.

c.843C>G; exon 10; p.Ile281Met (protein defect unclear)

I281M/1. This male patient (CaseE; Tuinenburg et al., 2018) was born 1992 to non-consanguineous parents. At age 6 he presented with bruising and a rash. Thrombocytopenia was subsequently found and he was diagnosed with ITP treated with steroids. Over the following year, it was noted that he had lymphadenopathy and up to seven of his glands in his head and neck area were enlarged. Three of these were biopsied and none were found to have any pathological findings. He was referred for immunological assessment and a provisional diagnosis of Autoimmune Lymphoproliferative Syndrome (ALPS) was made. He underwent 5 rituximab courses of treatment between the ages of 7-15 years old and was concomitantly commenced on intravenous immunoglobulin therapy which continued into adulthood. Intermittent episodes of neutropenia were also noted. At age 17 he was hospitalized whilst abroad with pneumonia and pleurisy. He continued to report recurrent sinopulmonary infections and remained thrombocytopenic. CT chest imaging confirmed bronchiectasis. His family history included an older sister with a history of alopecia areata and joint pains and his father died aged 47 from lymphoma. On further analysis of the patient’s previous results, he was noted to have no double negative T cell population and apoptosis assays and serum biomarkers for ALPS were normal with no genetic mutation identified. CD19 lymphopenia was noted, though he had received rituximab treatment. He had decreased percentage of IgM and switched memory B cells and increased percentage of transitional B cells. His diagnosis was revised to CVID, EUROClass $B^{+}smB^{-}Tr^{high}CD21^{low}$. He continues to have several sinopulmonary infections a year and has remained stable on immunoglobulin infusions. Western blotting of patient-derived neutrophils indicated slightly reduced p50 expression (Tuijnenburg et al., 2018).

c.851G>C; exon 10; p.Arg284Pro

R284P/1 and R284P/2. This male patient (R284P/1) was born in 2003. He presented at 16 years of age with a history of recurrent upper respiratory tract infections since three months of age, recurrent abdominal pain, diarrhea, and a recent weight loss of 9 kg. A celiac antibody panel was negative except for a low IgA. Subsequently, serum immunoglobulin measurements showed IgG of 5.86 g/l, IgA of <0.31 g/l and IgM of 0.29 g/l, all below the reference ranges for age. The patient was then seen in consultation for these findings. His physical examination did not reveal lymphadenopathy or hepatosplenomegaly. His lymphocyte subset analysis

showed normal numbers and percentages of T cells, T cell subsets, B cells and NK cells. However, B cell subset analysis demonstrated 92% naïve B cells and 2% switched memory B cells. His antibody response to Pneumovax® was suboptimal with positive responses to eight of 23 serotypes tested. A diagnosis of CVID was made and the patient responded well to immunoglobulin replacement therapy. The family history was significant for maternal hypothyroidism, paternal recurrent sinusitis and two healthy siblings.

A 407 gene primary immunodeficiency panel (Invitae™, San Francisco, CA, US) identified nine variants of uncertain significance including one in *NFKB1* (c.851G>C, p.Arg284Pro). Consequently, Sanger sequencing of *NFKB1* was carried out in the patient and his parents. This confirmed the patient's variant in *NFKB1* and that it was shared with his father (R284P/2).

c.856T>A; exon 10; p.Tyr286Asn

Y286N/1. This female patient was born in 1975 to unrelated parents. In her childhood she had frequent common colds but no pneumonia. At age 7 she had a tympanoplasty tube inserted in the left ear due to recurrent otitis media. When she became 25, her susceptibility to infections increased significantly. She suffered permanently from common colds, recurrent maxillary sinusitis and middle ear effusions. She also had splenomegaly and pangastritis, however with no evidence of *Helicobacter pylori*. After immunological evaluation at age 26, all immunoglobulin classes were found to be low (IgG 0.49 g/l, IgA <0.23 g/l, IgM 0.18 g/l). Laboratory findings also included low absolute B cell counts and low plasmablasts and slightly decreased CD8+ T cells and NK-cells. Multitest Merieux showed anergy. The diagnosis was CVID group Ia of the Freiburg classification or B^+ smB^- $CD21^{low}$ of the EURO Class. At age 26 she had cholecystitis and underwent cholecystectomy. At age 31, peripheral B cells were absent and she was reclassified as EURO Class B^-. Immunoglobulin replacement therapy was started at diagnosis. At this same age she developed monoarthritis of the left knee that evolved into a symmetric polyarthritis of the large joints. RF, CCP, and HLA-B27 were negative and no crystals or pathogens were found. A few months later, she developed chronic diarrhea. Colonoscopy revealed inflammatory and aphtous lesions in the cecum and the ascending colon. Histology was consistent with Crohn's disease. Immunosuppression with sulfasalazine, methotrexate and oral prednisolone was started. Two years later methotrexate was changed to adalimumab due to adverse effects. At age 35, repeated colonoscopy and histology showed no signs of Crohn's disease, even though diarrheal episodes persisted. At age 36, gastroscopy showed active *Helicobacter pylori* positive gastritis. In her late 30's she experienced recurrent esophageal candidiasis and herpes zoster infections on her back which continue until present. At the beginning of her 40's osteoporosis and psoriasis were diagnosed. At age 44 the patient presented with increased susceptibility to infections, including laringitis, maxillary sinusitis, esophageal candidiasis, herpes zoster and multiple HPV-like skin warts. A reduction of the steroid therapy from 10 mg to 7 mg is being attempted.

c.885G>C; exon 10; p.Trp295Cys

W295C/1. This male patient was born in 1956 to non-consanguineous parents. In his childhood he went through uneventful measles, rubella, varicella and mumps infections. He had a long history of recurrent bacterial bronchitis. When 27-years old, he had aspirin-induced thrombocytopenia. At age 34 he developed lymphocytic alveolitis and started treatment with low dose prednisone. At this time, he underwent immunological evaluation and IgG and IgA were found to be decreased (IgG 2.6 g/l, IgA 0.28 g/l, IgM 1.16 g/l). After further immunological evaluation, he was diagnosed with CVID Freiburg Class Ia, EURO Class B+ smB- Tr^{high} and immunoglobulin replacement therapy was started. At age 40 splenomegaly was observed and at age 43 he presented with lymphoproliferation of cervical lymphnodes from unknown

significance. In the context of his immunodeficiency, the patient also developed autoimmune sprue-like enteropathy, initially treated with cyclosporin and later with tacrolimus after cyclosporine-induced leukopenia. When 53, bronchiectasis was observed on his thorax CT scan. A sinobronchial syndrome was suspected and at age 54 he underwent sinus surgery with septoplasty. At the same age osteoporosis was diagnosed and two years later he had a femur fracture. He had idiopathic immune thrombocytopenia and recurrent autoimmune hemolytic anemia, treated with several cycles of rituximab since age 56. The patient developed liver cirrhosis and portal hypertension. When 62-years old he required hospitalization because of progressive ascites, an umbilical hernia and grade III esophageal varices, treated several times with esophageal banding. Nodular regenerative hyperplasia was suspected but not confirmed by histology. In addition, he had 12 kg of weight loss, associated with 6-month chronic diarrhea, which improved after change of tacrolimus to mycophenolate. During the hospitalization he had another episode of autoimmune hemolytic anemia. Serology revealed a past hepatitis B infection. Seven months later he had an incarceration of his umbilical hernia with subsequent necrosis of the intestinal mucosa and a loop ileostomy and enteral nutrition were required. He underwent transjugular intrahepatic portosystemic shunt placement. The patient also suffered from chronic kidney disease and corticosteroid-induced diabetes at age 62. He died at age 63 following a catheter sepsis.

c.1049A>G; exon 11; p.Tyr350Cys

Y350C/1 – Y350C/4. The four affected relatives of a German non-consanguineous CVID family were previously reported (Fliegauf et al., 2021). Western blot analyses of cells derived from patients 2 and 3 demonstrated reduced p105/p50 levels in both, PBMCs and EBV-immortalized B cells (Fliegauf et al., 2021).

c.1156G>A; exon 12; p.Gly386Arg (protein defect unclear)

G386R/1. This female Caucasian patient was born in 2009, with no family history of consanguinity. Her father has IgA-deficiency, vitiligo, pernicious anemia, brachial neuritis, recurrent pneumonia, bronchiectasis and gastric metaplasia; her mother has Crohn's disease; and her 12 year-old brother had recurrent infectious bronchitis up to 5 years of age and hypogammaglobulinemia. From the age of 3 years she had recurrent supurative otitis with persistent purulent rhinorrhea and 2 episodes of tonsillitis. At 6 years, adenoidectomy was performed with amygdalar reduction and placement of bilateral transtympanic tubes. Six months later, she had lost the right ear tube, restarting recurrent otitis media. At age 7, during a work-up for tube replacement, thrombopenia was detected. She developed abdominal pain accompanied by vaginal bleeding, frequent epistaxis and hematomas as well as habitual asthenia, which was aggravated with sports. A hematological study was performed, with a diagnosis of autoimmune thrombocytopenia (platelets between 18,000 and 100,000/mm^3). A decrease in the levels of IgG and IgA was detected and she was referred for immunological studies. Isoagglutinins were negative. An initial response to tetanus and anti-pneumococcal 23 vaccination was lost in less than a year. She was subsequently diagnosed with CVID. At 8 years and 11 months immunoglobulin replacement therapy was started, with clinical improvement and increase in the platelet count to 98,000/mm^3. She developed intermittent inguinal lymphadenopathy, usually increasing in size the week before intravenous immunoglobulin. Due to the persistence of the adenopathies, a lymph node biopsy was performed at 9 years of age with a final diagnosis of reactive lymphoid hyperplasia. At age 10, she started having episodes of dysarthria and paresthesia episodes. At 12 she was diagnosed with migraine with aura and began prophylactic amitriptyline. Immunological studies of B cell subpopulations between age 8 to 12 revealed a progressive decrease in the percentage of

switched memory cells and an increase in transitional B cells and an expansion of $CD21^{low}$ B cells. At 10 years, splenomegaly with an accessory spleen was detected. At 11 years, the frequency of respiratory infections and suppurative otitis increased and she had lymphadenopathies in other locations (axillary, retroperitoneal and paraaortic). Splenectomy was performed due to severe hypersplenism and doubt of an underlying lymphoma, leaving the accessory spleen. Histology revealed a reactive lymphoid follicular hyperplasia with expansion of CD4 + PD1 + T cells. Two months later she was admitted for febrile gastroenteritis with PCR in stool positive for *Salmonella*, *Giardia* and *Escherichia coli*, she was treated with good recovery effect. Following diagnosis of the *NFKB1* variant c.1156G>A (p.Gly386Arg), rapamycin treatment was started. Five months later, MRI showed a decrease in the chronic sinupathy with a stability in the size of the lymphadenopathies. A family study confirmed both, father and brother, carrying the same mutation.

<u>c.1214A>G; exon 13; p.Tyr405Cys</u>

Y405C/1. This female patient was born in 2002, has a history of chronic thrombocytopenia and systemic lupus erythematosus and has been managed primarily by rheumatology up to now. The thrombocytopenia responded to rituximab in 2019 after proving refractory to mycophenolate mofetil. She has systemic lupus erythematosus. Laboratory evaluation revealed normal immune globulins, normal complement C3, C4 of 17 mg/dL (normal 15-45 mg/dL), normal lymphocyte subsets (basic panel which includes CD3, CD8, CD19, CD56). Coombs test was positive. ANA was positive.

References

1. Anim M, Sogkas G, Schmidt G, Dubrowinskaja N, Witte T, Schmidt RE, Atschekzei F. Vulnerability to Meningococcal Disease in Immunodeficiency Due to a Novel Pathogenic Missense Variant in NFKB1. Front Immunol. 2021 Dec 24;12:767188. doi: 10.3389/fimmu.2021.767188. PMID: 35003082; PMCID: PMC8738076.
2. Christiansen M, Offersen R, Jensen JMB, Petersen MS, Larsen CS, Mogensen TH. Identification of Novel Genetic Variants in CVID Patients With Autoimmunity, Autoinflammation, or Malignancy. Front Immunol. 2020 Jan 27;10:3022. doi: 10.3389/fimmu.2019.03022. PMID: 32047491; PMCID: PMC6996488.
3. Duan L, Feanny S. Novel heterozygous NFKB1 mutation in a pediatric patient with cytopenias, splenomegaly, and lymphadenopathy. LymphoSign Journal 6(2). DOI:10.14785/lymphosign-2019-0006.
4. Fliegauf M, Krüger R, Steiner S, Hanitsch LG, Büchel S, Wahn V, von Bernuth H, Grimbacher B. A Pathogenic Missense Variant in NFKB1 Causes Common Variable Immunodeficiency Due to Detrimental Protein Damage. Front Immunol. 2021 Apr 27;12:621503. doi: 10.3389/fimmu.2021.621503. PMID: 33995346; PMCID: PMC8115018.
5. Govindarajan GV, Bhanumurthy L, Balasubramanian A, Ramanathan A. A Novel Mutation in the DNA Binding Domain of NFKB is Associated with Speckled Leukoplakia. Asian Pac J Cancer Prev. 2016;17(7):3627-9. PMID: 27510021.
6. Kaustio M, Haapaniemi E, Göös H, Hautala T, Park G, Syrjänen J, Einarsdottir E, Sahu B, Kilpinen S, Rounioja S, Fogarty CL, Glumoff V, Kulmala P, Katayama S, Tamene F, Trotta L, Morgunova E, Krjutškov K, Nurmi K, Eklund K, Lagerstedt A, Helminen M, Martelius T, Mustjoki S, Taipale J, Saarela J, Kere J, Varjosalo M, Seppänen M. Damaging heterozygous mutations in NFKB1 lead to diverse immunologic phenotypes. J Allergy Clin Immunol. 2017 Sep;140(3):782-796. doi: 10.1016/j.jaci.2016.10.054. Epub 2017 Jan 21. PMID: 28115215.
7. Li J, Lei WT, Zhang P, Rapaport F, Seeleuthner Y, Lyu B, Asano T, Rosain J, Hammadi B, Zhang Y, Pelham SJ, Spaan AN, Migaud M, Hum D, Bigio B, Chrabieh M, Béziat V, Bustamante J, Zhang SY, Jouanguy E, Boisson-Dupuis S, El Baghdadi J, Aimanianda V, Thoma K, Fliegauf M, Grimbacher B, Korganow AS, Saunders C, Rao VK, Uzel G, Freeman AF, Holland SM, Su HC, Cunningham-Rundles C, Fieschi C, Abel L, Puel A, Cobat A, Casanova JL, Zhang Q, Boisson B. Biochemically deleterious human NFKB1 variants underlie an autosomal dominant form of common variable immunodeficiency. J Exp Med. 2021 Nov 1;218(11):e20210566. doi: 10.1084/jem.20210566. Epub 2021 Sep 2. PMID: 34473196; PMCID: PMC8421261.
8. Lorenzini T, Fliegauf M, Klammer N, Frede N, Proietti M, Bulashevska A, Camacho-Ordonez N, Varjosalo M, Kinnunen M, de Vries E, van der Meer JWM, Ameratunga R, Roifman CM, Schejter YD, Kobbe R, Hautala T, Atschekzei F, Schmidt RE, Schröder C, Stepensky P, Shadur B, Pedroza LA, van der Flier M, Martínez-Gallo M, Gonzalez-Granado LI, Allende LM, Shcherbina A, Kuzmenko N, Zakharova V, Neves JF, Svec P, Fischer U, Ip W, Bartsch O, Barış S, Klein C, Geha R, Chou J, Alosaimi M, Weintraub L, Boztug K, Hirschmugl T, Dos Santos Vilela MM, Holzinger D, Seidl M, Lougaris V, Plebani A, Alsina L, Piquer-Gibert M, Deyà-Martínez A, Slade CA, Aghamohammadi A, Abolhassani H, Hammarström L, Kuismin O, Helminen M, Allen HL, Thaventhiran JE, Freeman AF, Cook M, Bakhtiar S, Christiansen M, Cunningham-Rundles C, Patel NC, Rae W, Niehues T, Brauer N, Syrjänen J, Seppänen MRJ, Burns SO, Tuijnenburg P, Kuijpers TW; NIHR BioResource, Warnatz K, Grimbacher B; NIHR BioResource. Characterization of the clinical and immunologic phenotype and management of 157 individuals with 56 distinct heterozygous NFKB1 mutations. J Allergy Clin Immunol. 2020 Oct;146(4):901-911. doi: 10.1016/j.jaci.2019.11.051. Epub 2020 Apr 9. PMID: 32278790.
9. Rojas-Restrepo J, Caballero-Oteyza A, Huebscher K, Haberstroh H, Fliegauf M, Keller B, Kobbe R, Warnatz K, Ehl S, Proietti M, Grimbacher B. Establishing the Molecular Diagnoses in a Cohort of 291 Patients With Predominantly Antibody Deficiency by Targeted Next-Generation Sequencing: Experience From a Monocentric Study. Front. Immunol., 17 December 2021 | https://doi.org/10.3389/fimmu.2021.786516.
10. Schröder C, Sogkas G, Fliegauf M, Dörk T, Liu D, Hanitsch LG, Steiner S, Scheibenbogen C, Jacobs R, Grimbacher B, Schmidt RE, Atschekzei F. Late-Onset Antibody Deficiency Due to

Monoallelic Alterations in NFKB1. Front Immunol. 2019 Nov 14;10:2618. doi: 10.3389/fimmu.2019.02618. PMID: 31803180; PMCID: PMC6871540.

11. Tuijnenburg P, Lango Allen H, Burns SO, Greene D, Jansen MH, Staples E, Stephens J, Carss KJ, Biasci D, Baxendale H, Thomas M, Chandra A, Kiani-Alikhan S, Longhurst HJ, Seneviratne SL, Oksenhendler E, Simeoni I, de Bree GJ, Tool ATJ, van Leeuwen EMM, Ebberink EHTM, Meijer AB, Tuna S, Whitehorn D, Brown M, Turro E, Thrasher AJ, Smith KGC, Thaventhiran JE, Kuijpers TW; NIHR BioResource–Rare Diseases Consortium. Loss-of-function nuclear factor κB subunit 1 (NFKB1) variants are the most common monogenic cause of common variable immunodeficiency in Europeans. J Allergy Clin Immunol. 2018 Oct;142(4):1285-1296. doi: 10.1016/j.jaci.2018.01.039. Epub 2018 Mar 2. PMID: 29477724; PMCID: PMC6148345.

12. Yang D, Zhang W, Liang J, Ma K, Chen P, Lu D, Hao W. Single cell whole genome sequencing reveals that NFKB1 mutation affects radiotherapy sensitivity in cervical cancer. Oncotarget. 2017 Dec 21;9(7):7332-7340. doi: 10.18632/oncotarget.23587. PMID: 29484114; PMCID: PMC5800906.
